# Supplementary material for: Ampere-level reduction of pure nitrate by electron-deficient Ru with K+ ions repelling effect
Source: Nat Commun. 2024 Dec 30;15:10877. doi: 10.1038/s41467-024-55230-w (PMC11685401; doi:10.1038/s41467-024-55230-w)
Supplement: Supplementary file 1 — Supplementary Information [file 41467_2024_55230_MOESM1_ESM.pdf]

Ampere-level reduction of pure nitrate by electron-deficient Ru with K^+ ions repelling effect

Authors: Shi-Nan Zhang†, Peng Gao†, Qian-Yu Liu, Zhao Zhang, Bing-Liang Leng, Jie-Sheng Chen and Xin-Hao Li*

Affiliations:

School of Chemistry and Chemical Engineering, Frontiers Science Center for Transformative Molecules, Shanghai Jiao Tong University, Shanghai 200240, P. R. China.

†These authors contributed equally: Shi-Nan Zhang, Peng Gao

*Correspondence to: xinhaoli@sjtu.edu.cn (Xin-Hao Li)

Table of Contents

Figures S1-S40 5

Figure S1. LSV curves of the a-Ru/NC electrode during continuous scanning. ...5

Figure S2. ESTEM-DF images of the 2D-Ru/NC catalyst 6

Figure S3. False-color ESTEM-DF images of the 2D-Ru/NC catalyst. 7

Figure S4. ESTEM-DF images and corresponding ESTEM-SEimages of the 2D-Ru/NC sample. 8

Figure S5 ESTEM-DF images and the relative Ru metal Z-contrast intensities of the 2D-Ru/NC catalyst 9

Figure S6. Characterizations of np-Ru/NC catalyst 10

Figure S7. BET test of bare NC, np-Ru/NC and 2D-Ru/NC samples 11

Figure S8. Ru *3p* and Cl *2p* XPS spectra of 2D-Ru/NC and a-Ru/NC samples. . 12

Figure S9. C *1s* and N *1s* XPS spectra of 2D-Ru/NC, a-Ru/NC, and bare NC samples 13

Figure S10. Wavelet transform for the k^3-weighted EXAFS spectra of np-Ru/NC sample 14

Figure S11. k^3-weighted Ru K-edge EXAFS spectra of 2D-Ru/NC sample and corresponding curve-fitting results with various fitting models 15

Figure S12. k^3-weighted Ru K-edge k-space oscillations of 2D-Ru/NC sample and corresponding curve-fitting with various fitting models. 16

Figure S13. k^3-weighted Ru K-edge EXAFS spectra and k-space oscillations of 2D-Ru/NC sample and corresponding curve-fitting of Cl. 17

Figure S14. k^3-weighted Ru K-edge k-space oscillations and corresponding curve-fitting results of Ru foil, np-Ru/NC, and RuO_2. 18

Figure S15. Calculation models and Bader charge for each Ru in np-Ru/NC and 2D-Ru/NC model 19

Figure S16. UPS spectra in the secondary electron cut-off and onset energy of 2D-Ru/NC, np-Ru/NC, and np-Ru/C catalysts. 20

Figure S17. LSV curves of 2D-Ru/NC in 1M KNO_3 and 0.5 M K_2SO_4. 21

Figure S18. LSV curves of a-Ru/NC and 2D-Ru/NC catalyst in 1M KNO_3 22

Figure S19. Calibration curves of ammonia by ^{1}H NMR analysis and Nessler method..23

Figure S20. NO_3RR performance of bare NC, np-Ru/C, np-Ru/NC, and 2D-Ru/NC catalysts in 1 M KNO_3 at −1.1 V vs. RHE. 24

Figure S21. Quantitation of N_2, H_2, NO_2^- and N_2H_4..25

Figure S22 NO_3RR performance of 2D-Ru/NC catalysts in 1 M KNO_3 under various potentials 26

Figure S23. NO_3RR performance of 2D-Ru/NC catalysts in various concentions of KNO_3 ranging from 0.001 M to 1 M...27

Figure S24. NH_3 yield rates and FE values of 2D-Ru/NC catalyst in various nitrate solution..28

Figure S25. i-t curves of 12 runs of electrocatalytic NO_3RR at −1.1 V vs RHE in 1 M KNO_3 solution over a reused 2D-Ru/NC electrode....................................29

Figure S26. XRD patterns of fresh and used 2D-Ru/NC samples......................30

Figure S27. TEM images and size distributions of Ru metal of fresh and used 2D-Ru/NC catalyst.. 31

Figure S28. C *1s*, N *1s* and Ru *3p* XPS spectra of fresh and used 2D-Ru/NC samples...32

Figure S29. I-t curves of 2D-Ru/NC catalysts in 1 M KNO_3 at −1.1 V vs. RHE and corresponding pH values of cathode electrolyte..33

Figure S30. Potential-dependent *in situ* Raman spectra of interfacial water on np-Ru/NC electrode surface during NO_3RR process. 34

Figure S31. *In situ* Raman spectra of interfacial water on 2D-Ru/NC and np-Ru/NC electrode surface at −1.4 V vs. Ag/AgCl. 35

Figure S32. Representative snapshots of pre-adsorbed various ions on the Ru, Ru-0.02, and Ru-0.04 model surface at different times during AIMD simulation process...36

Figure S33. Average distance of pre-adsorbed K^+ ion to catalyst surface...........37

Figure S34 Distance of pre-adsorbed NO_3^- ion to the catalyst surface. 38

Figure S35. Distance of pre-adsorbed OH^- ion to the catalyst surface................39

Figure S36. Distance of pre-adsorbed Li^+, Na^+, and Cs^+ ions to the catalyst surface. 40

Figure S37. LSV curves of 2D-Ru/NC and np-Ru/NC based electrodes in 0.5 M KOH.......41

Figure S38. Chronopotentiometry curves and FE values of NO_3RR over 2D-Ru/NC electrodes at 1 A cm^{-2} in 1 M KNO_3 solution.......42

Figure S39. Cyclic voltammetry curve for Ag/AgCl electrode calibration.......423

Figure S40. Photographs of in situ FTIR and Raman set-up.......424

Table S1-S8.......45

Table S1. Ru contents of different samples obtained by ICP-AES results.......45

Table S2. Structure parameters derived from the EXAFS simulation of 2D-Ru/NC simple with different fitting models.......46

Table S3. Structure parameters derived from the EXAFS simulation of various samples.......47

Table S4. Gibbs free energy change of each step in the proposed mechanism of NO_3RR on Ru and Ru-0.04 model.......48

Table S5. NO_3RR performance of the 2D-Ru/NC and state-of-art electrocatalysts in neutral condition.......49

Table S6. NO_3RR performance of the 2D-Ru/NC and state-of-art Ru-based electrocatalysts.......50

Table S7. The proportions of the three types of interfacial water on 2D-Ru/NC and np-Ru/NC surface at different potentials.......51

Table S8. NO_3RR performance under ampere-lever current density over 2D-Ru/NC electrode and other reported Ru-based electrocatalysts.......52

References.......53

Figures S1-S40

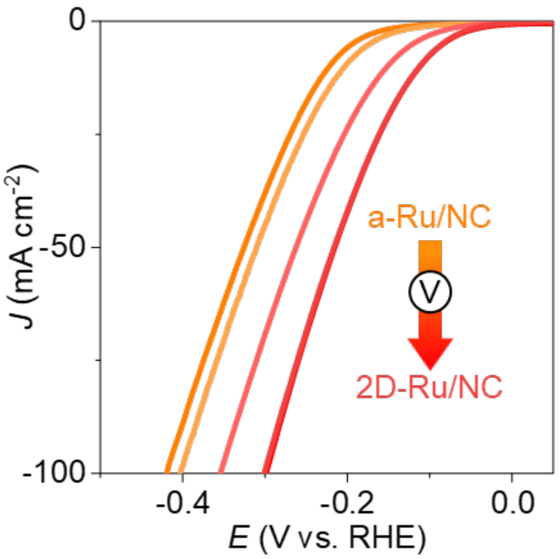

Supplementary Figure 1. LSV curves of the a-Ru/NC electrode obtained during continuous scanning in Ar-saturated 0.5 M H_2SO_4. During the continuous LSV scanning, the current density initially increased and then reached steady state. The gradually enhanced current densities indicate the electric current-driven reduction to form Ru metal along the NC support, representing the formation of 2D-Ru/NC.

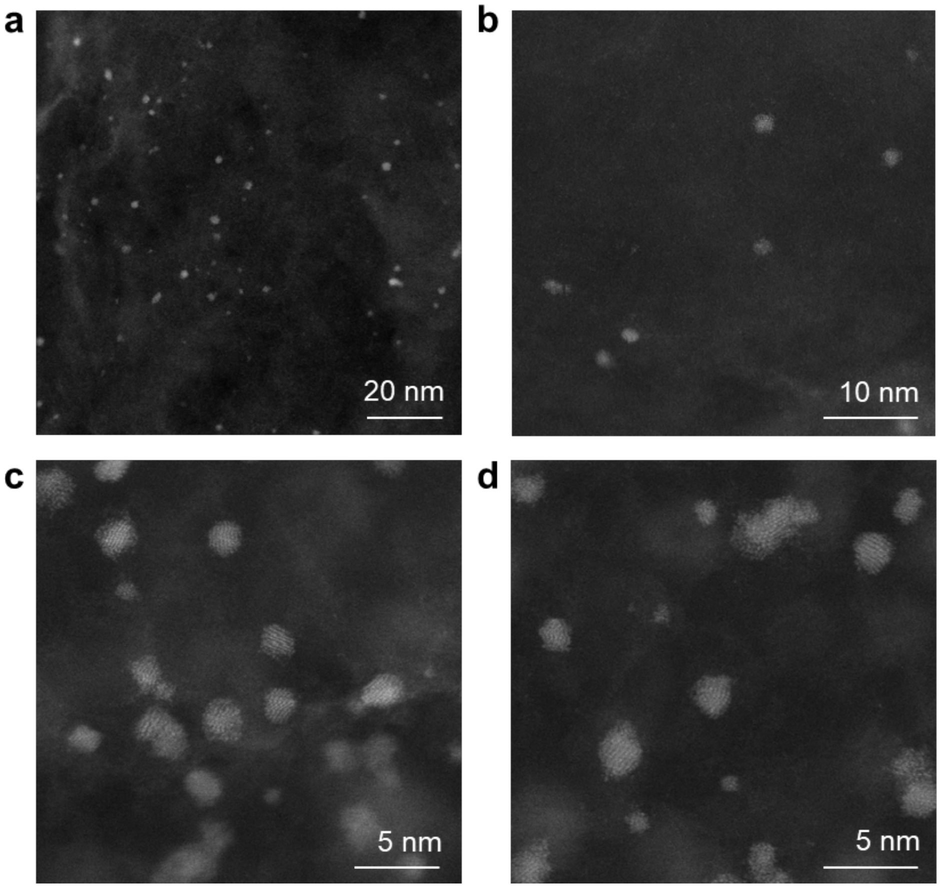

Supplementary Figure 2. a-d ESTEM-DF images of the 2D-Ru/NC catalyst. After the electrochemical reduction process, the metallic Ru can be observed along the whole NC support by the difference of Z-contrast, conforming the successful reduction of Ru.

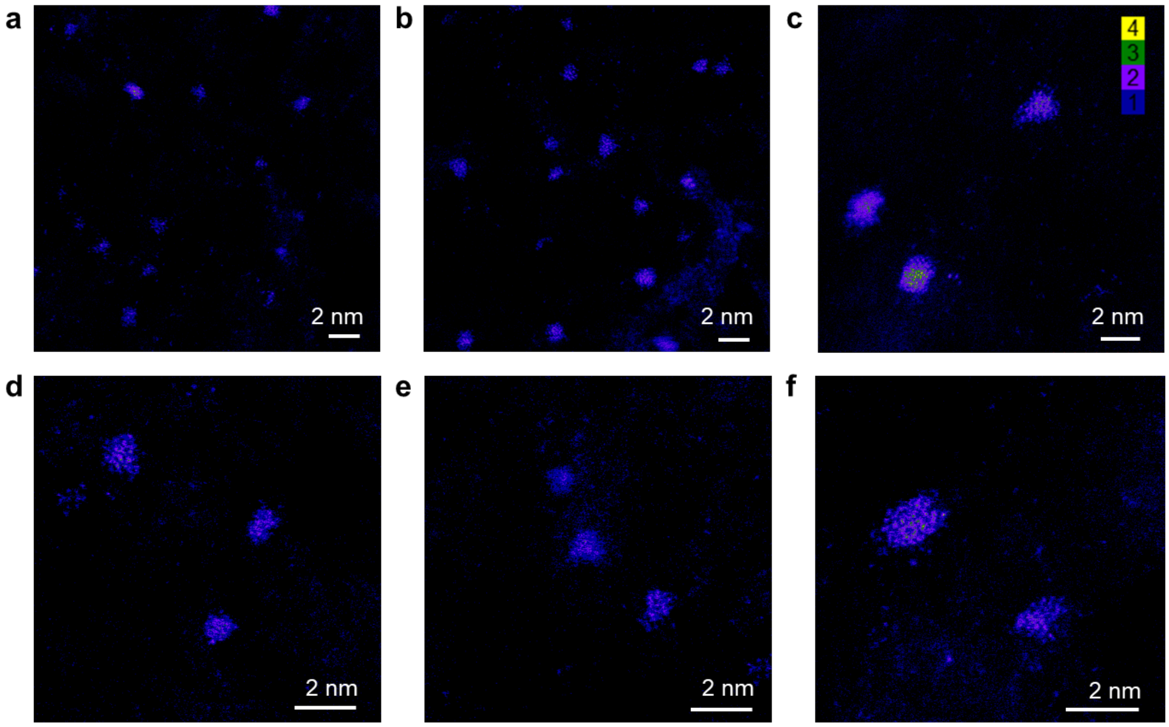

Supplementary Figure 3. a-f False-color ESTEM-DF images of the 2D-Ru/NC catalyst. We revealed the 2D structure of Ru metals by comparing the difference of DF intensity between Ru metals and single atomic Ru, as there is a monotonic relationship between the DF intensity and atomic layer numbers or thickness.[1,2] First, according to the line profiles in the ESTEM-DF images, we can define the intensity of atomic Ru as a basic intensity unit after subtracting the intensity of NC support. Then, we measured the DF intensity of corresponding Ru metals along the line profiles and defined the ratio of DF intensity between Ru metals and atomic Ru as the relative intensity. Finally, the relative intensities of the Ru metals were visualized as the false-color ESTEM-DF images, as present in Figure 1c and S3. All the formed Ru metal presented similar layer numbers (1~2), confirming the 2D structure of the Ru metals in 2D-Ru/NC catalyst.

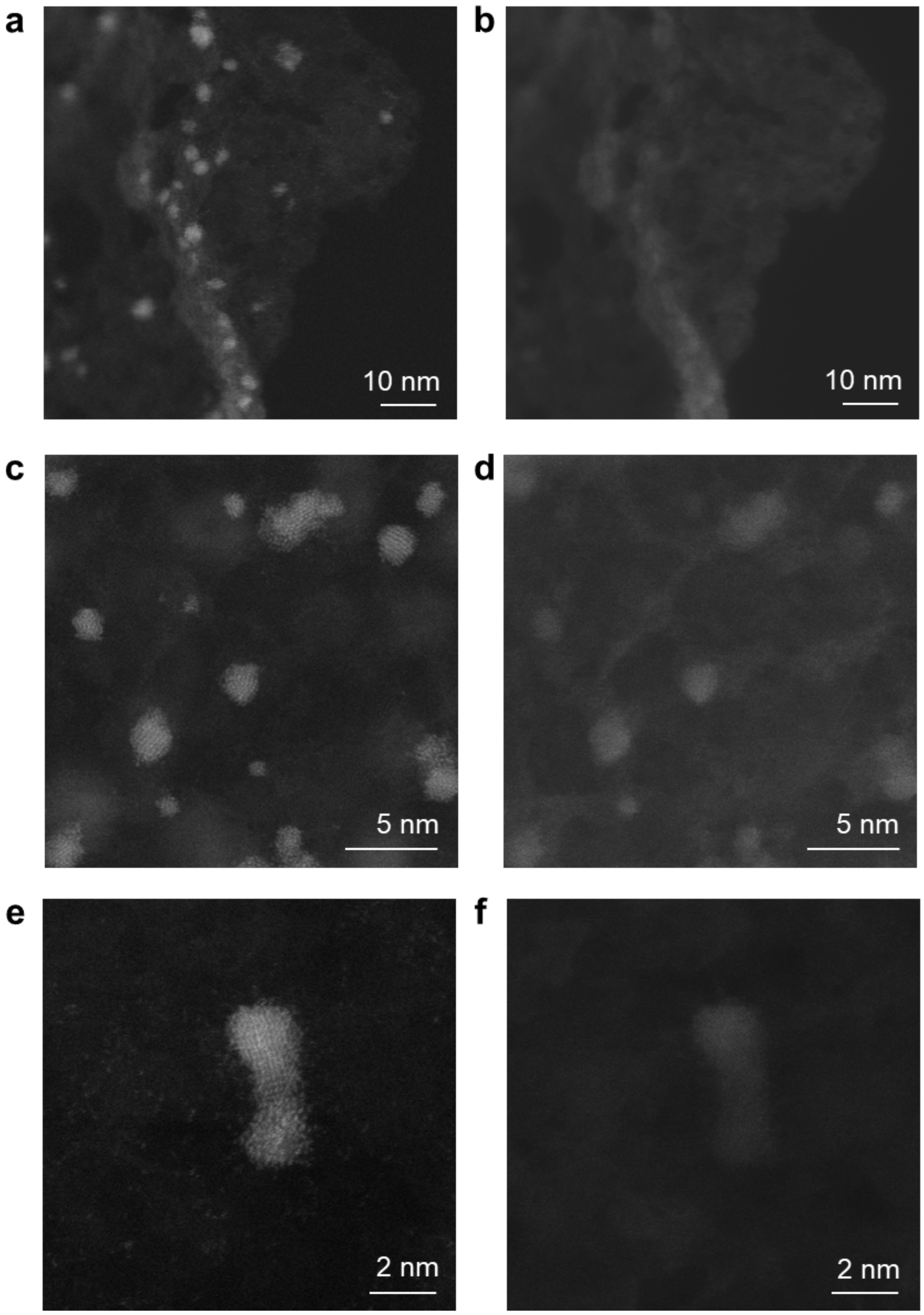

Supplementary Figure 4. a-f ESTEM-DF images (**a**, **c**, **e**) and corresponding secondary-electron (ESTEM-SE) images (**b**, **d**, **f**) of the 2D-Ru/NC sample. In ESTEM-SE images, the Ru metals were usually observed as noticeably brighter areas. The little difference in Z-contrast between Ru metals and NC support in **b**, **d**, and **f** as compared to **a**, **d**, and **e**, respectively, confirmed the ultra-thin of Ru metals in the 2D-Ru/NC sample.

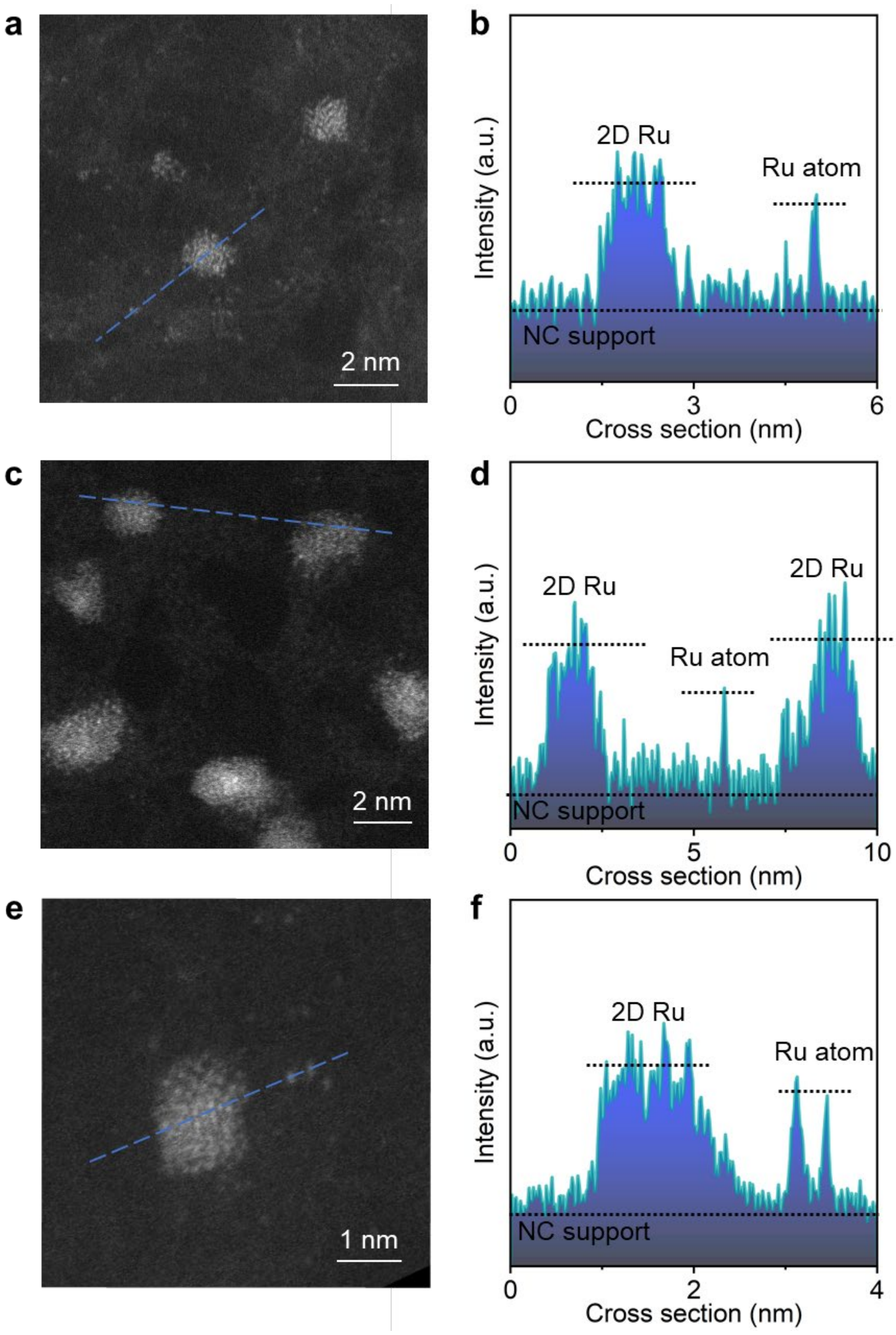

Supplementary Figure 5 a-f ESTEM-DF images (**a**, **c**, **e**) and the relative Ru metal Z-contrast intensities compared to atomic Ru (**b**, **d**, **f**) of the 2D-Ru/NC catalyst. The DF intensity of the Ru metal is comparable to that of atomic Ru, indicating the thin structure of Ru metal in 2D-Ru/NC catalyst.

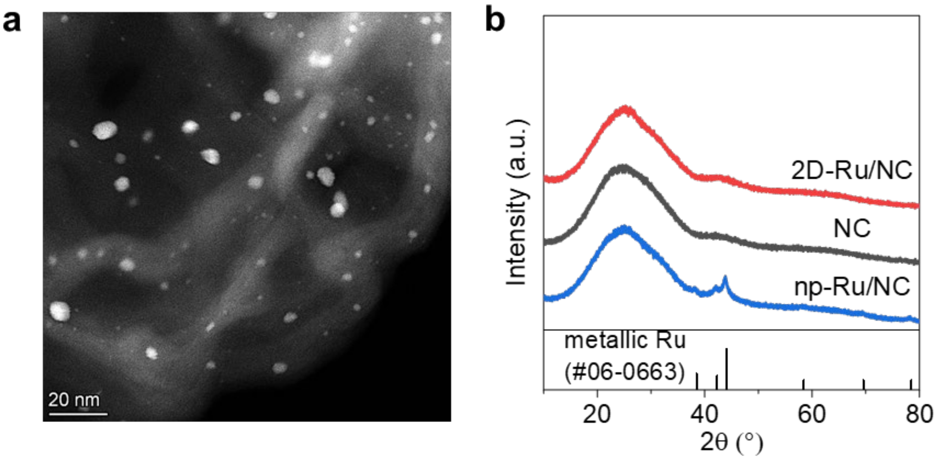

Supplementary Figure 6. a TEM image of the typical np-Ru/NC catalyst. **b** XRD pattern of typical 2D-Ru/NC, NC and np-Ru/NC samples. The control sample NC supported Ru nanoparticles (np-Ru/NC) was prepared via widely used wet impregnation methods with comparable Ru loadings to 2D-Ru/NC sample (Table S1). Besides the different shape (**a**), the np-Ru/NC sample exhibits two typical XRD peaks (**b**) of Ru metals at 42.1° and 43.8°, which are not observable in the XRD pattern of 2D-Ru/NC sample, indicating the formation of nanocrystals with unregular spherical shape in the np-Ru/NC sample.

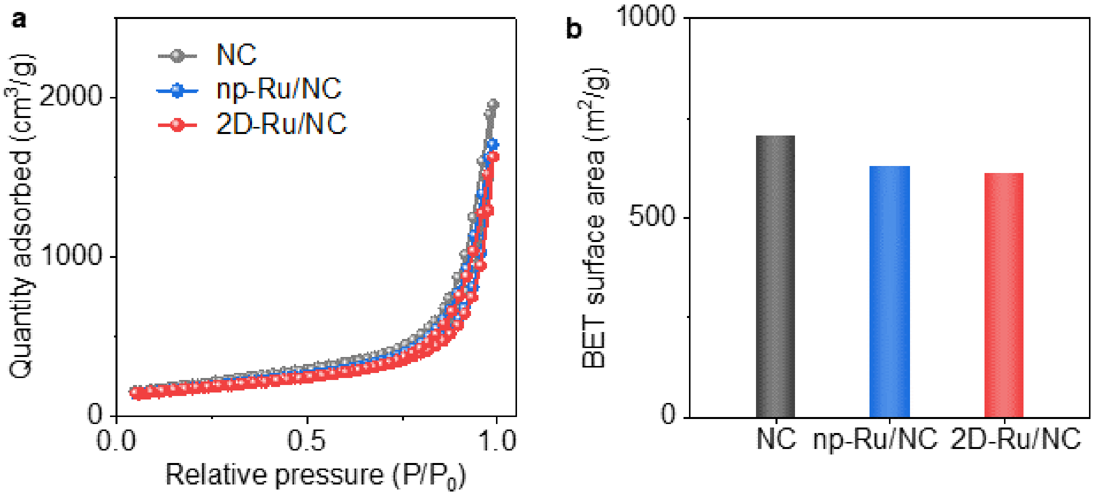

Supplementary Figure 7. a-b The N_2 adsorption isotherm curves (**a**) and Brunauer-Emmett-Teller (BET) surface area (**b**) of bare NC, np-Ru/NC and 2D-Ru/NC samples. The BET surface areas of 2D-Ru/NC and np-Ru/NC are very close, which could exclude the significant effect of specific surface area on the final performance of NO_3RR.

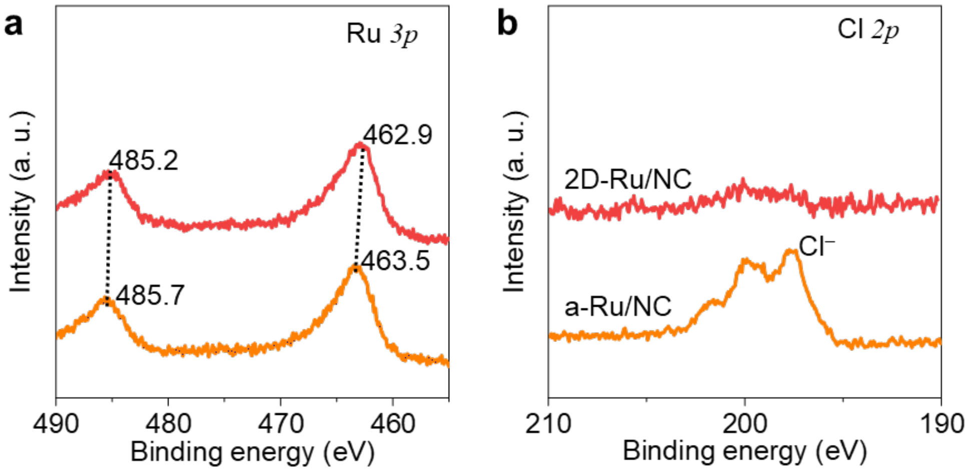

Supplementary Figure 8. a-b Ru *3p* (**a**) and Cl *2p* (**b**) XPS spectra of 2D-Ru/NC and a-Ru/NC samples. The negative shift in Ru *3p* peak (**a**) confirms the formation of Ru metals during the electrochemical reduction process. The negligible Cl *2p* XPS peak (**b**) of 2D-Ru/NC sample reveals the successful removal of Cl^{-} to release the Ru surface.

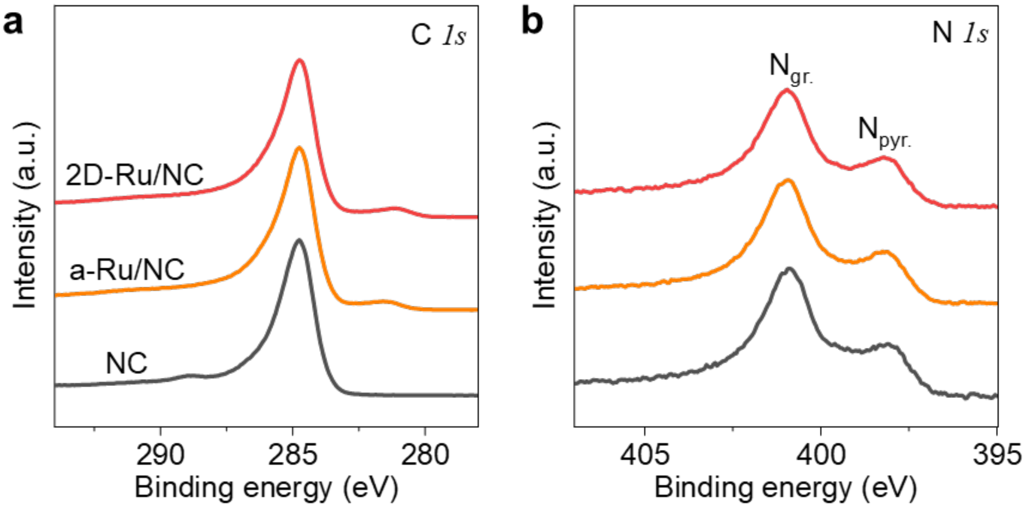

Supplementary Figure 9. a-b C *1s* (**a**) and N *1s* (**b**) XPS spectra of 2D-Ru/NC, a-Ru/NC, and bare NC samples. The C *1s* and N *1s* XPS spectra confirm that the electrochemical reduction process did not significantly change the component of NC support.

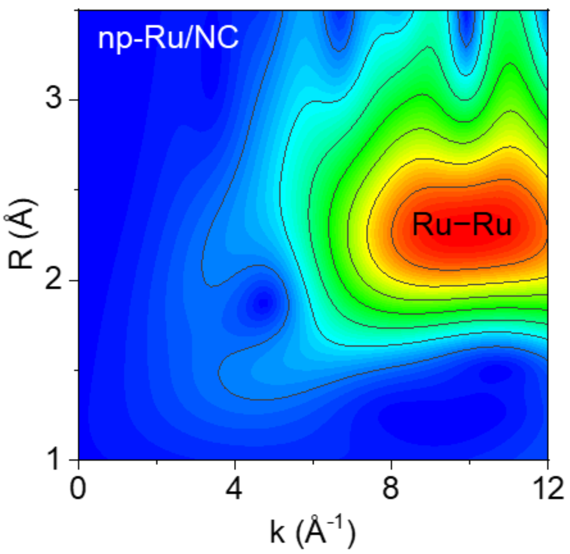

Supplementary Figure 10. Wavelet transform for the k^3-weighted EXAFS spectra of np-Ru/NC sample. The obvious area of Ru–Ru bond, similar to that of Ru foil, confirmed the formation of Ru metal in np-Ru/NC sample.

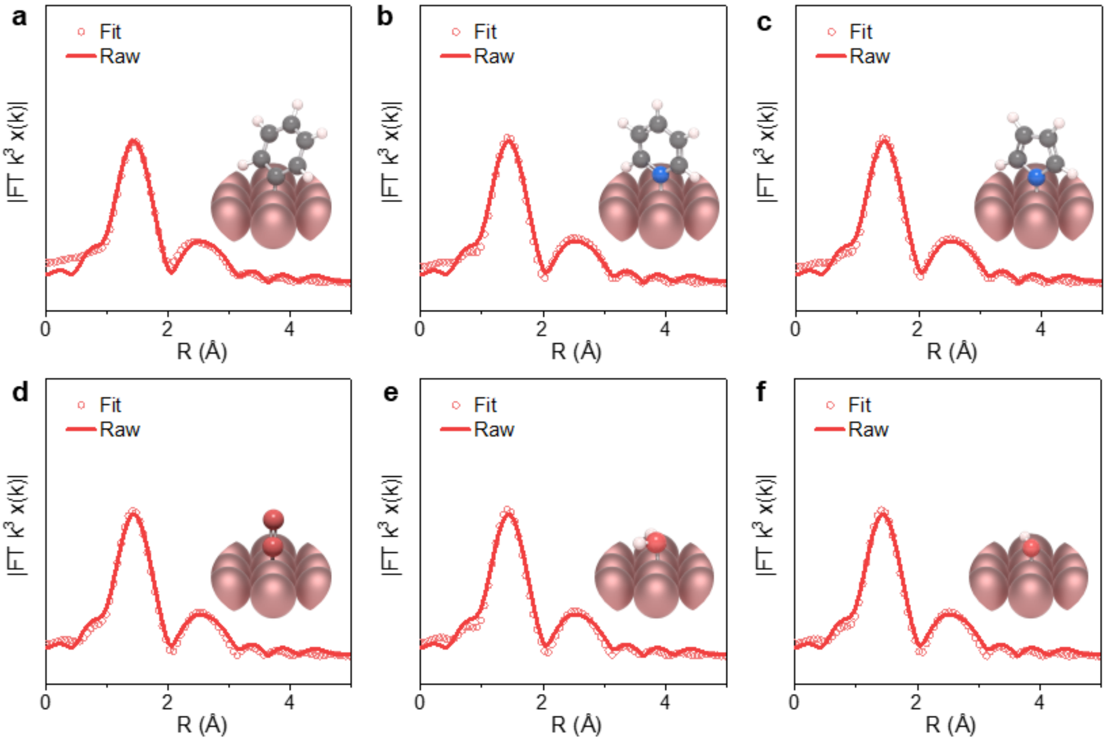

Supplementary Figure 11. a-f The k^3-weighted Ru K-edge EXAFS spectra (solid line) of 2D-Ru/NC sample and corresponding curve-fitting (circle line) of graphite carbon (**a**), pyridine nitrogen (**b**), pyrrole nitrogen (**c**), O_2 (**d**), H_2O (**e**), and hydroxyl (**h**). Insets: fitting models (Ru, Red brown; O, red; N, blue; H, white; C, grey). To identify the chemical structure of the Ru nanolayer in 2D-Ru/NC catalyst, we use graphite carbon, pyridine nitrogen, and pyrrole nitrogen to simulate the defects as possible coordinating cites in NC support; we also use O_2, H_2O, and hydroxyl to simulate the typical adsorbed oxygen species on the Ru metal surface. All these models gave us satisfied fitting results (Figure S10-11) and (Table S2), proving the 2D structure of Ru with arbitrary C, N, or O-based adsorbates in 2D-Ru/NC catalyst.

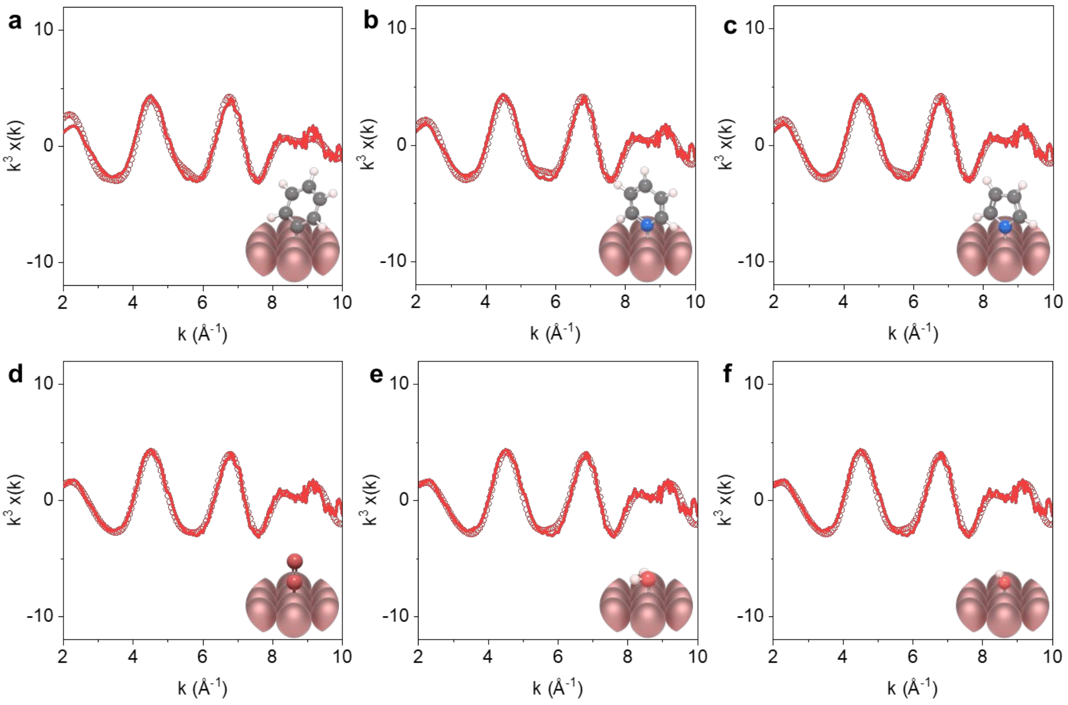

Supplementary Figure 12. a-f The k^3-weighted Ru K-edge k-space oscillations (solid line) of 2D-Ru/NC sample and corresponding curve-fitting (circle line) of graphite carbon (**a**), pyridine nitrogen (**b**), pyrrole nitrogen (**c**), O_2 (**d**), H_2O (**e**), and hydroxyl (**h**). Insets: fitting models (Ru, Red brown; O, red; N, blue; H, white; C, grey). The well-fitting results indicated that all these species are possible adsorbates on the nanolayer Ru of 2D-Ru/NC.

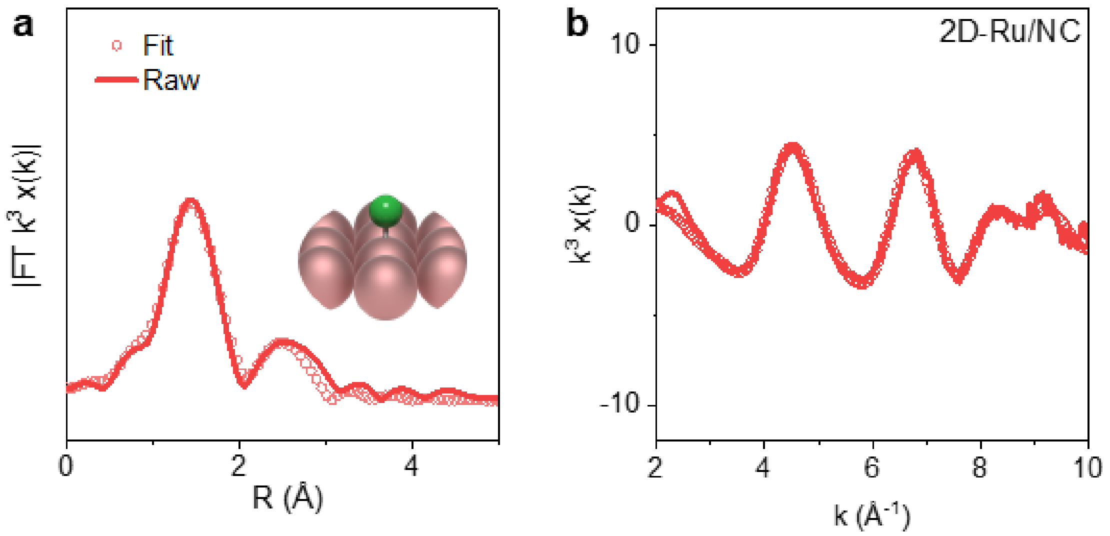

Supplementary Figure 13. a The k^3-weighted Ru K-edge EXAFS spectra (solid line) of 2D-Ru/NC sample and corresponding curve-fitting of Cl (circle line). Insets: fitting model (Ru, Red brown; Cl, green). **b** The k^3-weighted Ru K-edge k-space oscillations (solid line) and corresponding curve-fitting of Cl (circle line). The misfits between the raw data of 2D-Ru/NC and fitting results (Figure S12) with unacceptable absorption edge offset value (ΔE, −27.19 eV) (Table S2) prove that Cl is not suitable as the possible counter ion for curve fitting. In other words, Cl^- ions have been successfully removed from Ru surface (Figure S7b) and do not adsorbed on the Ru surface in 2D-Ru/NC catalyst.

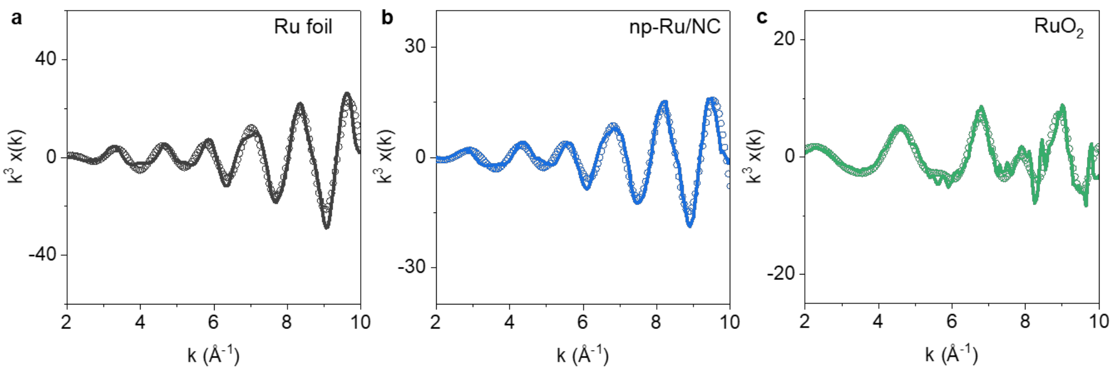

Supplementary Figure 14. a-c The k^3-weighted Ru K-edge k-space oscillations (solid line) and corresponding curve-fitting results (circle line) of Ru foil (**a**), np-Ru/NC (**b**), and RuO_2 (**c**).

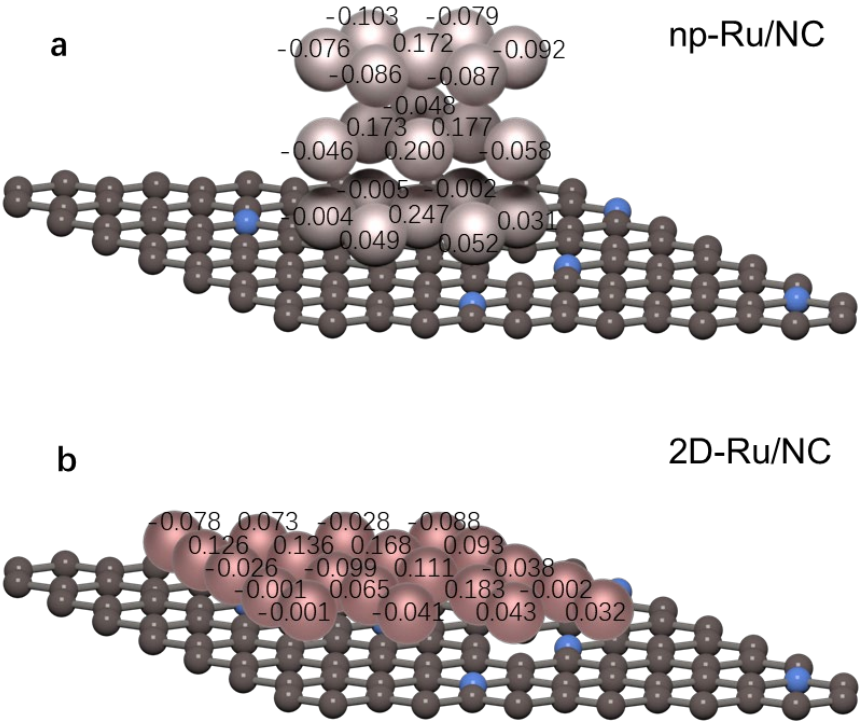

Supplementary Figure 15. a-b Calculation models and Bader charge for each Ru in np-Ru/NC (**a**) and 2D-Ru/NC (**b**) model**.** To solely consider the influence of the Ru metal structure, we used the same NC support and kept the number of Ru atoms the same in both models. According to the calculation results, the average Bader charge of Ru in 2D-Ru/NC model is 0.04, which is twice as much as that (0.02) in np-Ru/NC**,** indicating an enhanced electron-deficiency of Ru in 2D-Ru/NC

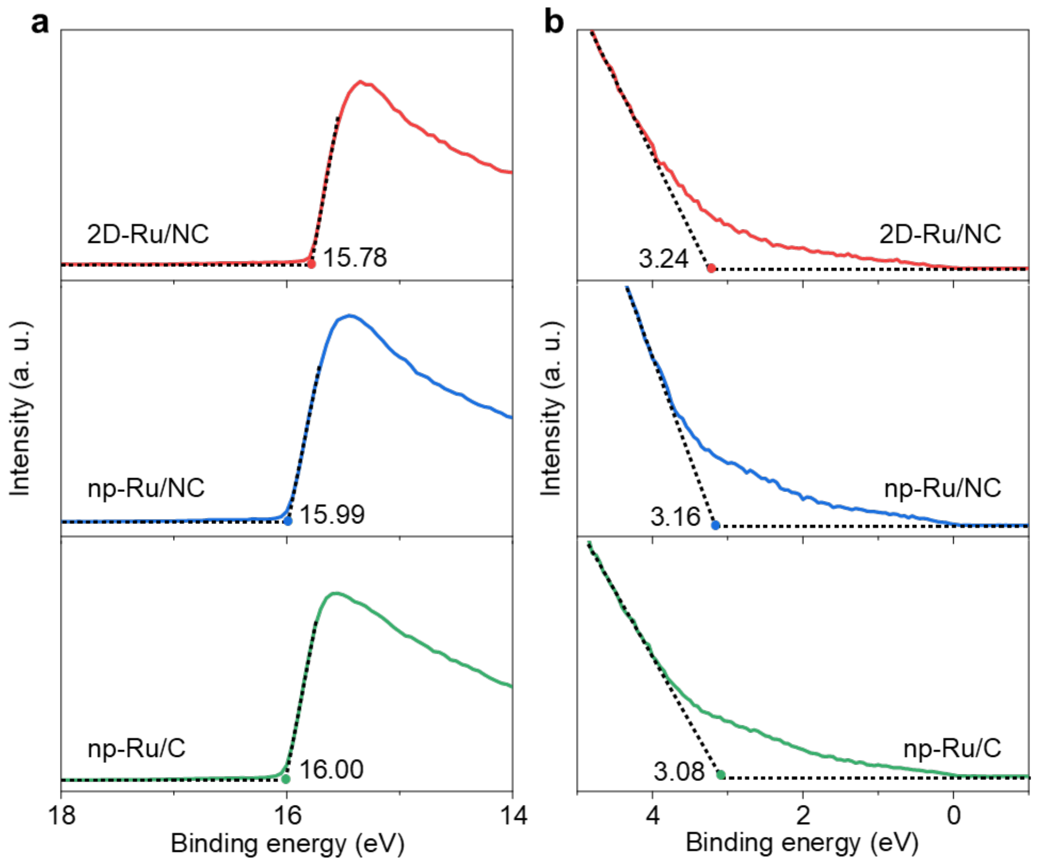

Supplementary Figure 16. a-b UPS spectra in the secondary electron cut-off (Ecut-off) (**a**) and onset (Ei) energy (**b**) of 2D-Ru/NC, np-Ru/NC, and np-Ru/C catalysts. The work functions (Φ), as calculated by the equation of $\Phi = 21.21\text{eV} - (E_{cut\text{-}off} - E_i)$, are 8.67, 8.38, and 8.29 eV for 2D-Ru/NC, np-Ru/NC, and np-Ru/C catalysts, respectively.

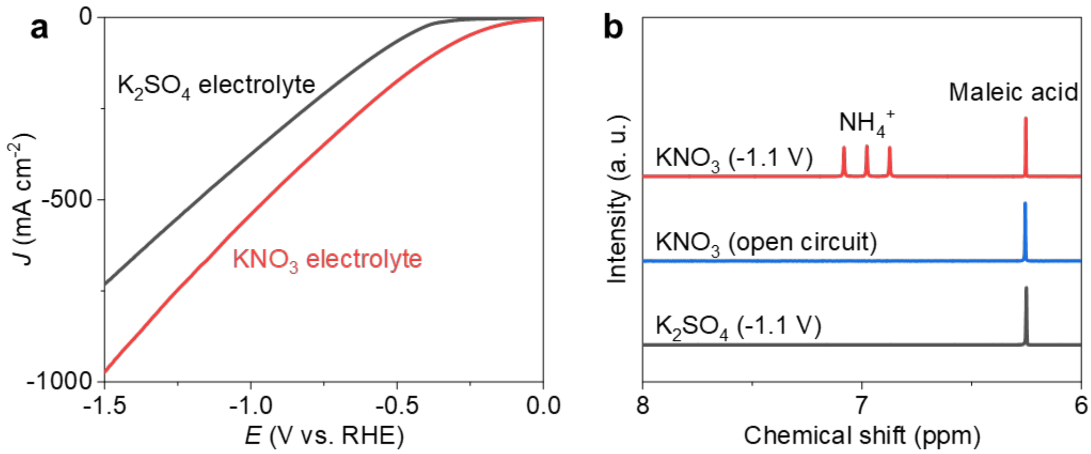

Supplementary Figure 17. a LSV curves of 2D-Ru/NC catalyst in 1M KNO_3 and 0.5 M K_2SO_4. **b** ^{1}H NMR spectra of the electrolyte after corresponding potentiostatic tests. The markedly increased NO_3RR current densities of the 2D-Ru/NC in 1 M KNO_3 (**a**), as compared with the measured HER current density in 0.5 M K_2SO_4, directly demonstrate the preference of NO_3RR on the 2D-Ru/NC electrocatalyst. There was no generation of ammonia for the blank experiments (**b**), indicating that the ammonia was directly produced from the electrocatalytic NO_3RR process.

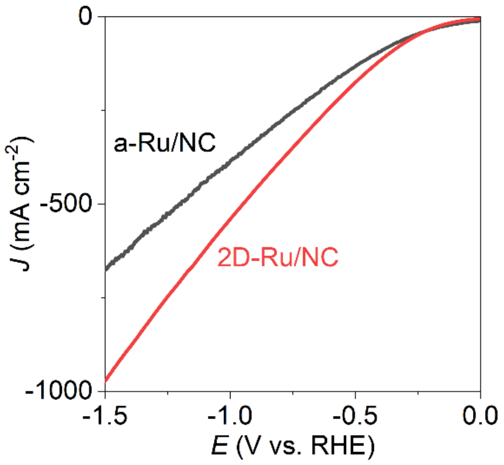

Supplementary Figure 18. LSV curves of a-Ru/NC and 2D-Ru/NC catalyst in 1M KNO_3. The significantly enhanced current densities of 2D-Ru/NC as compared to those of the pre-catalyst a-Ru/NC indicated the vital role of the as-formed 2D Ru sheets in NO_3RR process.

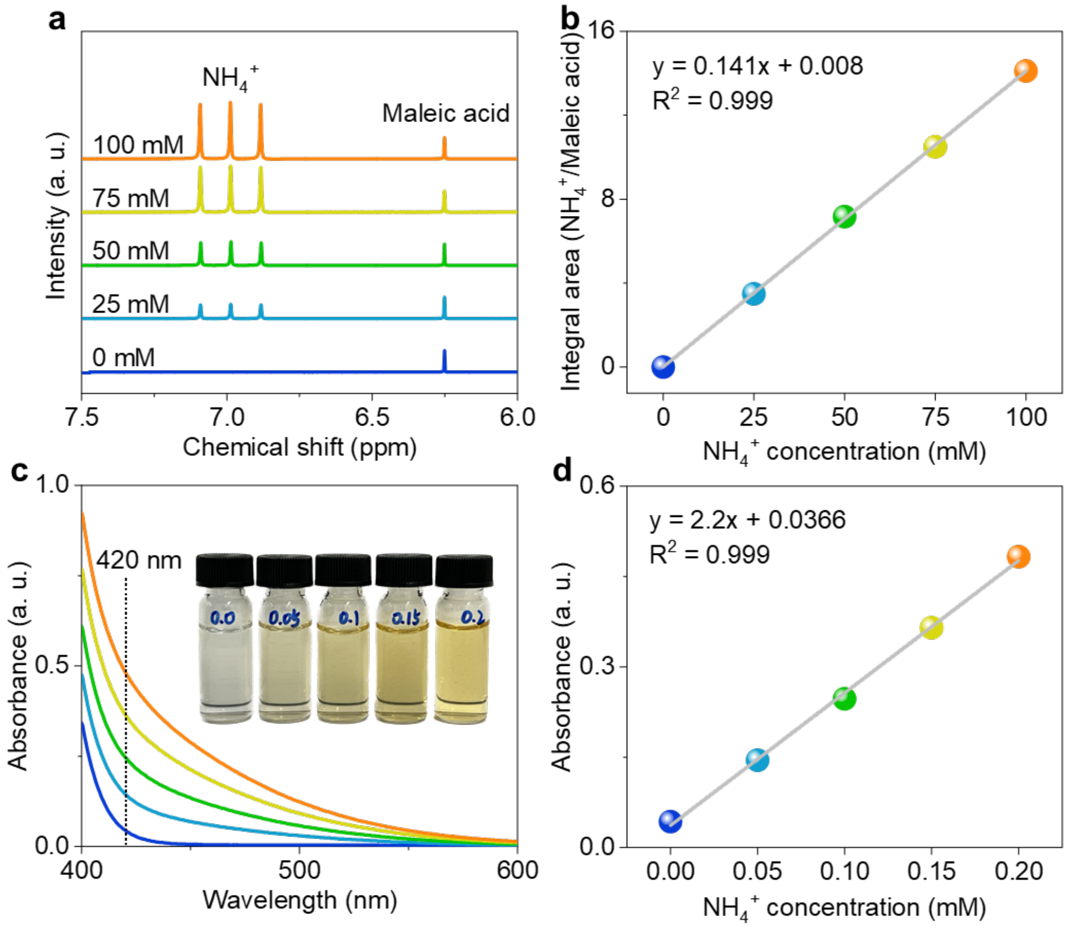

Supplementary Figure 19. Calibration curves of ammonia by ^{1}H NMR analysis and Nessler method. **a-b** ^{1}H NMR spectra of $^{14}NH_4^+$ at different concentrations using maleic acid as an external standard (**a**) and the corresponding standard curve (**b**). The $^{14}NH_4^+$ showed triple peaks at $\delta = 6.88$, 6.99, and 7.09 ppm and the maleic acid showed a single peak at $\delta = 6.25$ ppm. **c-d** UV-vis absorption spectra with different concentrations of ammonia using Nessler method (**c**) and the corresponding standard curve (**d**). Inset: digital image of the solutions with different NH_4^+ concentrations tested by Nessler method. The calibration curves of both ^{1}H NMR analysis and Nessler method show great linearity. Moreover, the ammonia yields quantified through ^{1}H NMR analysis and the Nessler method are in great agreement, confirming the reliability of our results.

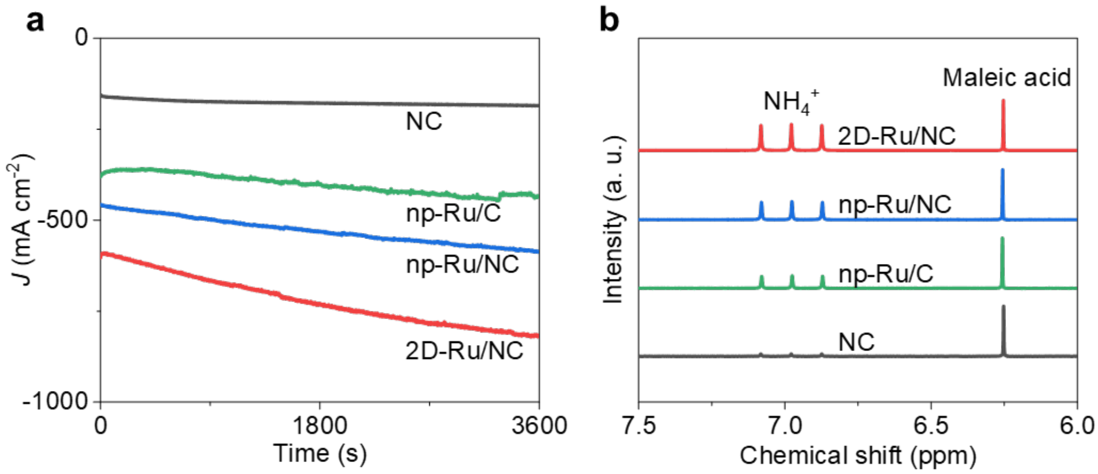

Supplementary Figure 20. a i-t curves of bare NC, np-Ru/C, np-Ru/NC, and 2D-Ru/NC catalysts in 1 M KNO_3 at −1.1 V vs. RHE. **b** ^{1}H NMR spectra of the electrolyte after the corresponding potentiostatic tests for various catalysts. According to the current density at fixed potential and ammonia yields calculated from the ^{1}H NMR results, 2D Ru/NC catalyst shows remarkable superiority for electrocatalytic NO_3RR over NC, np-Ru/C, and np-Ru/NC catalysts.

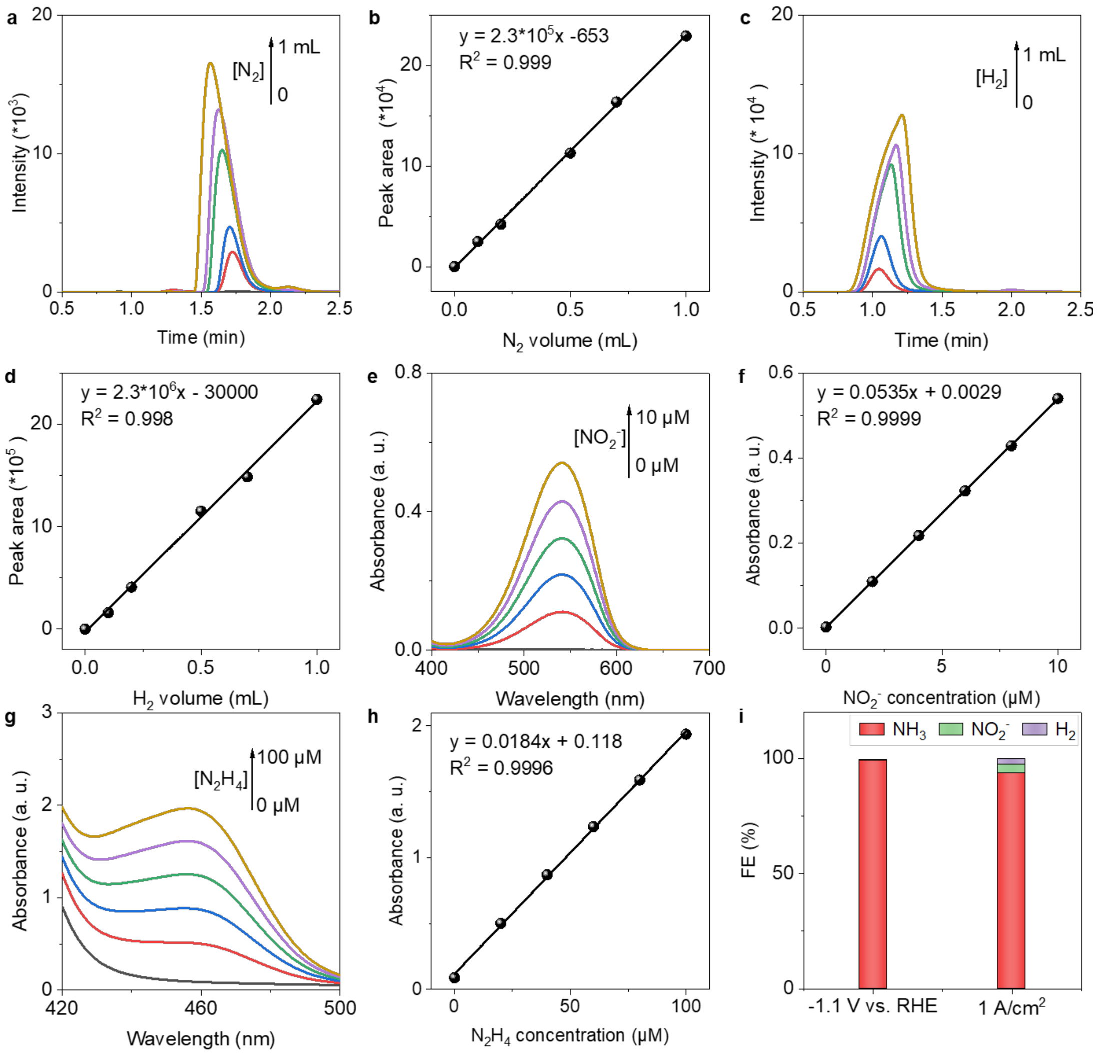

Supplementary Figure 21. Quantitation of N_2, H_2, NO_2^- and N_2H_4. **a-b** GC chromatograms (**a**) with different volumes of N_2 and corresponding standard curve (**b**). **c-d** GC chromatogram (**c**) with different volumes of H_2 and corresponding standard curve (**d**). **e-f** UV-vis absorption spectra with different concentrations of NO_2^- using Griess method (**e**) and the corresponding standard curve (**f**). **g-h** UV-vis absorption spectra with different concentrations of N_2H_4 using Watt-Chrisp method method (**g**) and the corresponding standard curve (**h**). **i** FE values of 2D-Ru/NC electrode for NO_3RR in 1 M KNO_3 at –1.1 V vs. RHE. After the potentiostatic test at –1.1 V vs. RHE, the 2D-Ru/NC catalyst achieved high FE value for NH_3 (> 99%) and negligible FE value for NO_2^- (~0.2%), while no gaseous products and N_2H_4 were detected. Moreover, the 2D-Ru/NC catalyst achieved a high FE value (94%) for NH_3 under ampere level current density, with a small amount of NO_2^- (FE value: ~4%) and H_2 (FE value: ~2%).

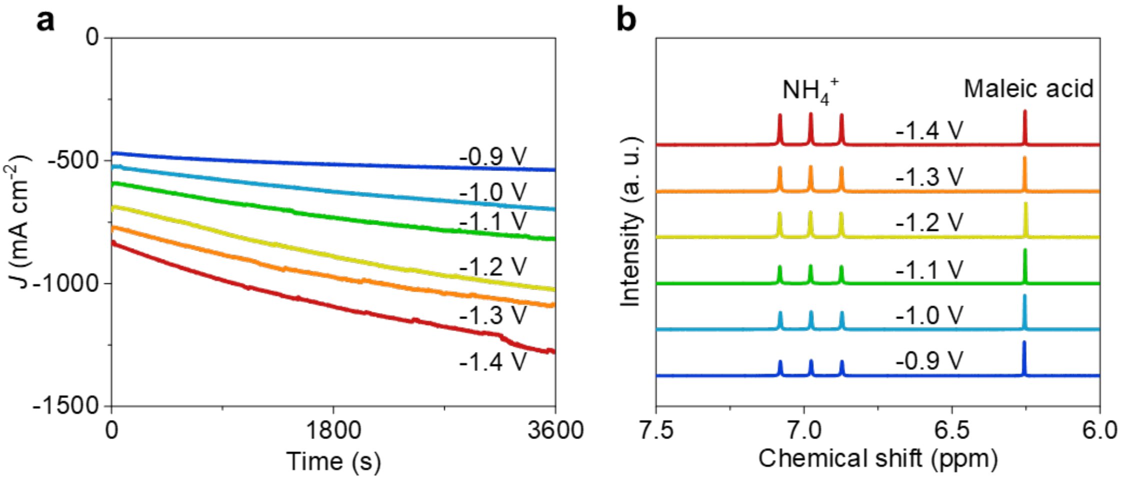

Supplementary Figure 22 a i-t curves of 2D-Ru/NC catalysts in 1 M KNO_3 under various potentials. **b** ^1H NMR spectra of the electrolyte after the corresponding potentiostatic tests under various potentials. Within a wide voltage range (−0.9 to −1.4 V vs. RHE), the NH_3 yield of 2D-Ru/NC catalyst increased as the increased voltage, accompanied by well-maintained FE values (>92%). At −1.1 V vs. RHE, the 2D-Ru/NC catalyst gave the highest FE value (> 99%), with high NH_3 yield (55.4 mg cm^{-2} h^{-1})

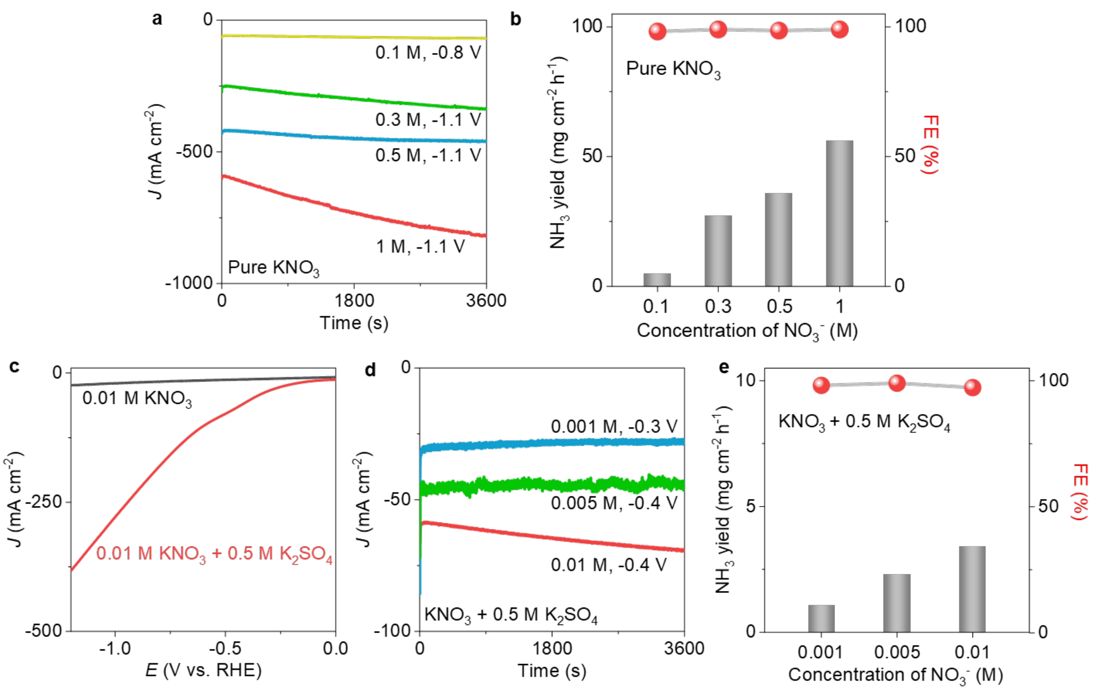

Supplementary Figure 23. a-b i-t curves (**a**) and corresponding NH_3 yield and FE values (**b**) of 2D-Ru/NC catalysts in pure KNO_3 solution ranging from 0.1 M to 1 M. **c** LSV curves of 2D-Ru/NC catalyst in electrolyte containing 0.01 M KNO_3 and electrolyte containing 0.01 M KNO_3 and 0.5 M K_2SO_4. **d-e** i-t curves (**d**) and corresponding NH_3 yield and FE values (**e**) of 2D-Ru/NC catalysts in a mixed solution of 0.5 M K_2SO_4 and KNO_3, where the concentration of KNO_3 ranges from 0.001 M to 0.01 M.

We tested the performance of our 2D-Ru/NC electrode in pure nitrate solutions ranging from 0.1 M to 1 M with Faradaic efficiencies between 98–99% to ammonia (**a-b**). When the nitrate concentration is further reduced, the reaction current becomes too low (**c**) due to the insufficient electrolyte in the solution. Thus, by adding K_2SO_4 as supporting electrolyte, high Faradaic efficiency (97–99%) to ammonia can still be achieved at ultra-low nitrate concentrations (0.001–0.01 M), with NH_3 yield from 1.1 to 3.4 mg cm^{-2} h^{-1} (**d-e**).

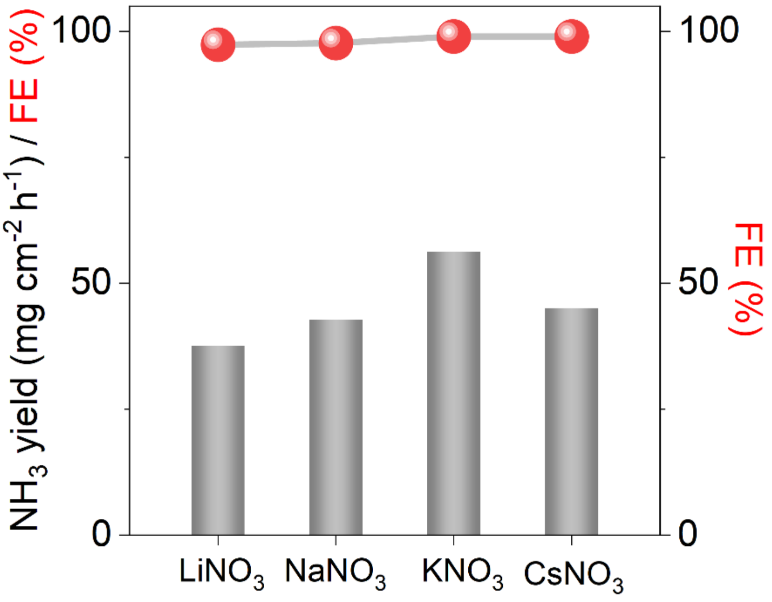

Supplementary Figure 24. NH_3 yield rates and FE values of 2D-Ru/NC catalyst in various nitrate solution (1 M NO_3^-) at -1.1 V vs. RHE. Under fixed conditions, the KNO_3 solution gave the highest NH_3 yield for NO_3RR. Thus. we choose K^+ ions as the main focus in this study.

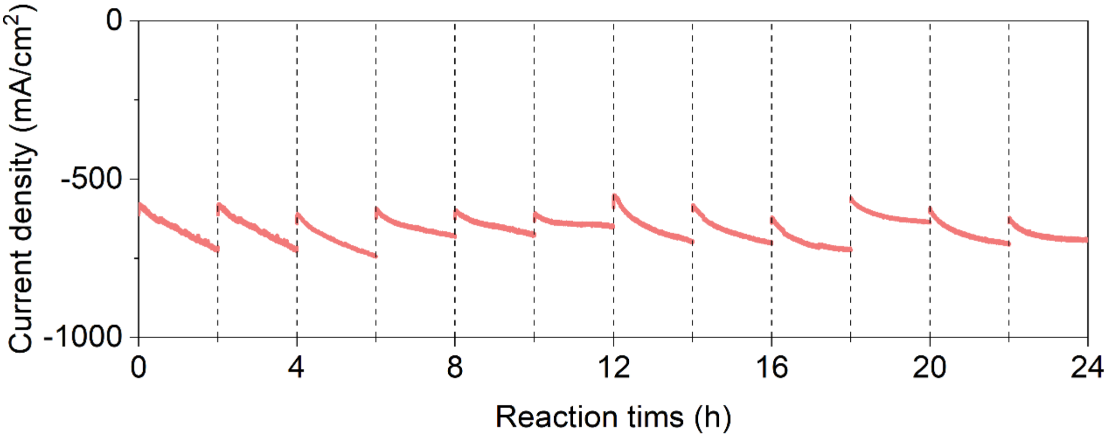

Supplementary Figure 25. i-t curves of 12 runs of electrocatalytic NO_3RR at −1.1 V vs RHE in 1 M KNO_3 solution over a reused 2D-Ru/NC electrode. During the 12 consecutive runs, the current density in the i-t curves kept the identical increased trend and the NH_3 yields and Faradaic efficiencies (Figure 3d) showed no obvious decay, indicating the durability of 2D-Ru/NC electrode.

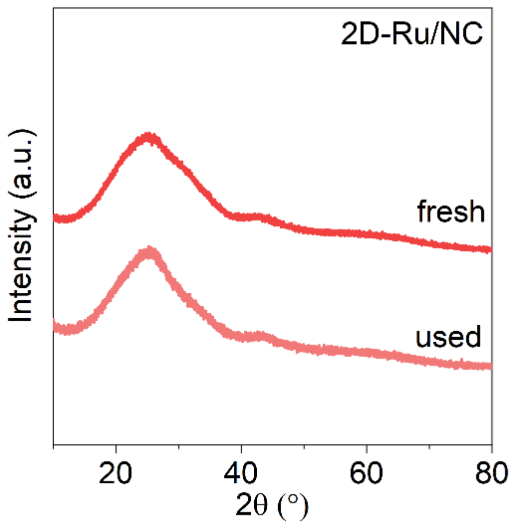

Supplementary Figure 26. XRD patterns of fresh and used 2D-Ru/NC samples. The similar XRD patterns indicate the stability of crystallinity of 2D-Ru/NC catalyst during NO_3RR process.

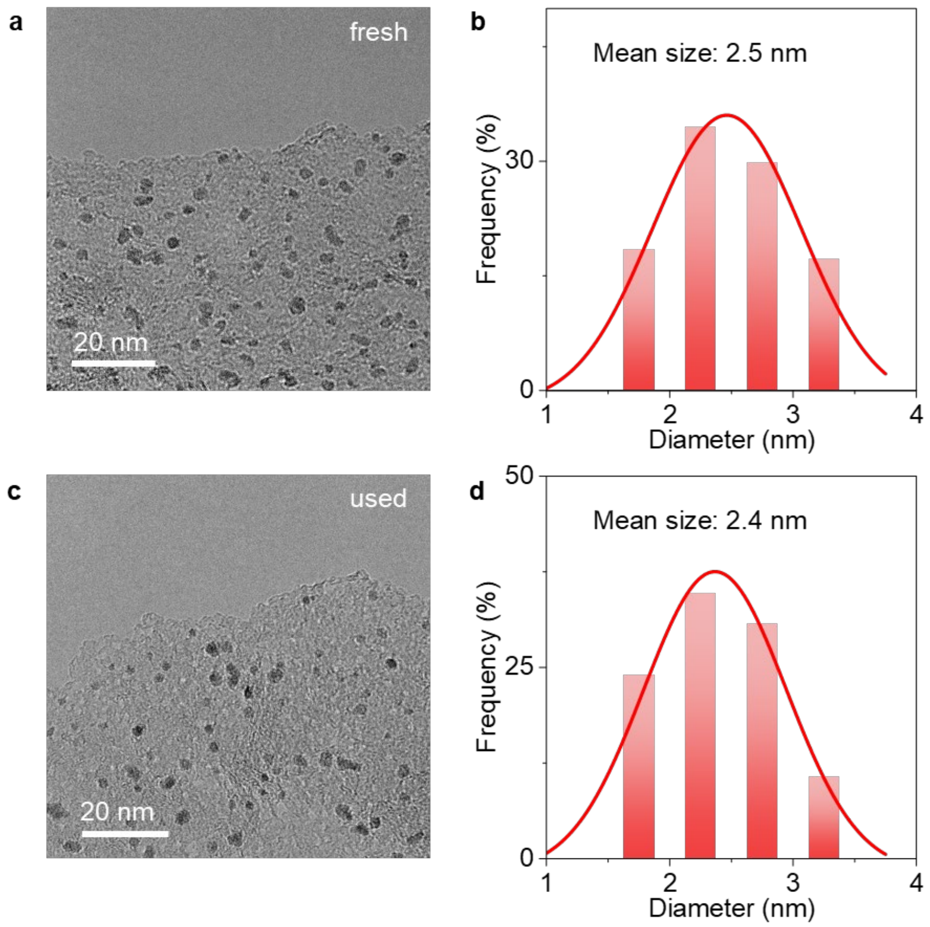

Supplementary Figure 27. a-d TEM images and size distributions of Ru metal of fresh (**a**-**b**) and used (**c**-**d**) 2D-Ru/NC catalyst. The similar size distributions of Ru metal indicate the morphological stability of 2D-Ru/NC catalyst during NO_3RR process, which is attributed to the strong interaction between the 2D Ru and the 2D NC support to keep from possible aggregation or leaching.

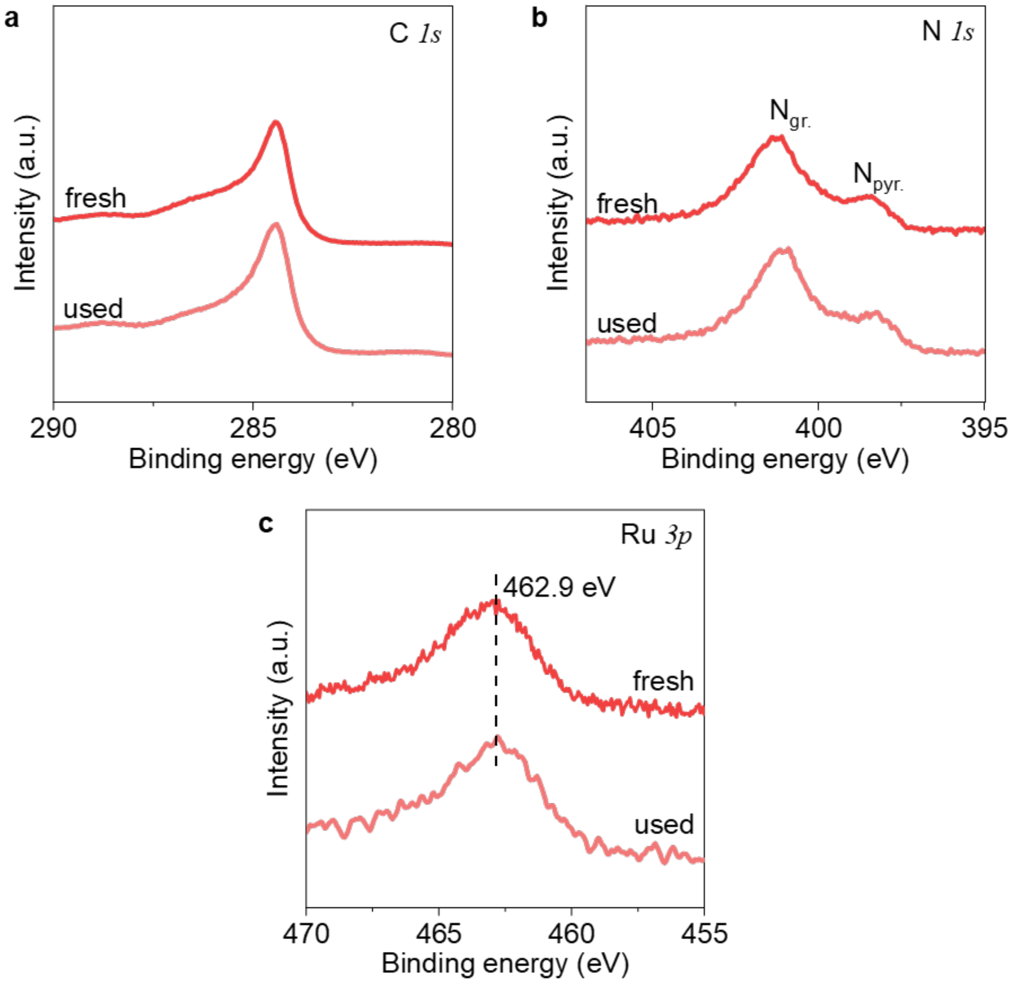

Supplementary Figure 28. a-c C *1s* (**a**), N *1s* (**b**) and Ru *3p* (**c**) XPS spectra of fresh and used 2D-Ru/NC samples. The XPS spectra of the used 2D-Ru/NC catalysts indicate the well-maintained chemical composition during the electrocatalytic reactions.

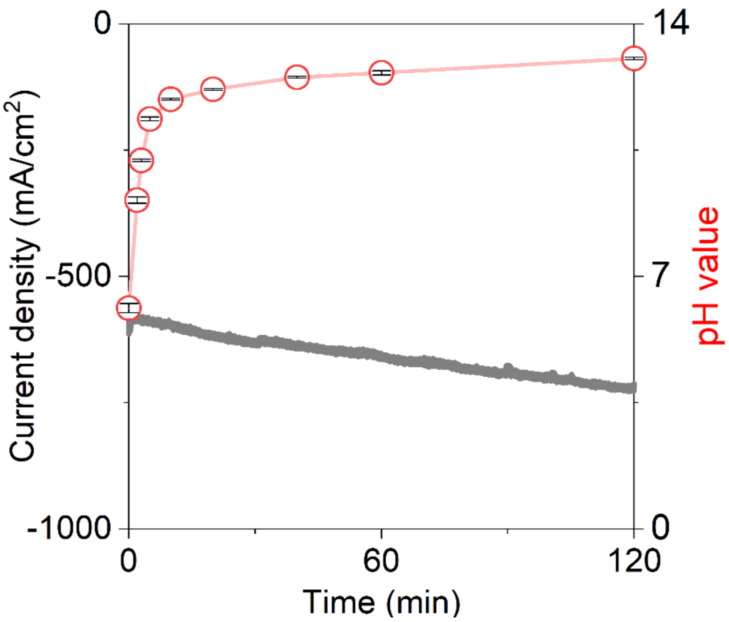

Supplementary Figure 29. i-t curves of 2D-Ru/NC catalysts in 1 M KNO_3 at −1.1 V vs. RHE and corresponding pH values of cathode electrolyte. The NO_3RR process is always accompanied by the formation of OH^- to accelerate the NO_3RR process due to the change in pH value of the electrolyte. Indeed, the pH value of the cathode electrolyte becomes as high as 10.2 within 3 minutes and finally to around 13.0 after two hours during the potentiostatic test at −1.1 V vs. RHE. The error bars were the relative standard deviations obtained by three repeated measurements.

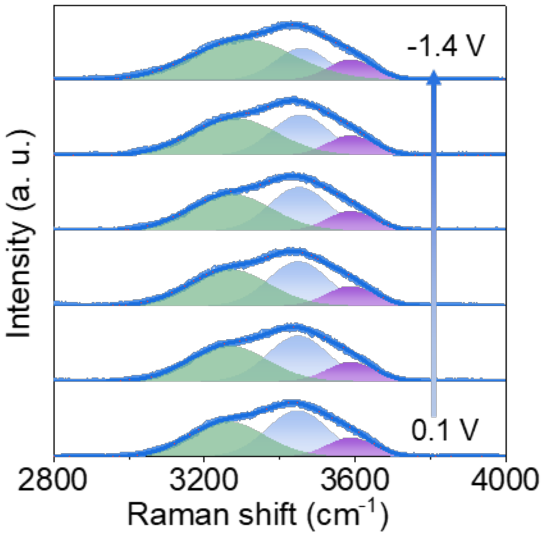

Supplementary Figure 30. Potential-dependent in situ Raman spectra of interfacial water on np-Ru/NC electrode surface during NO_3RR process. Gaussian fits of three O–H stretching models of 4-HB·H2O, 2-HB·H2O, and K^+·H_2O are shown in green, blue, and purple, respectively.

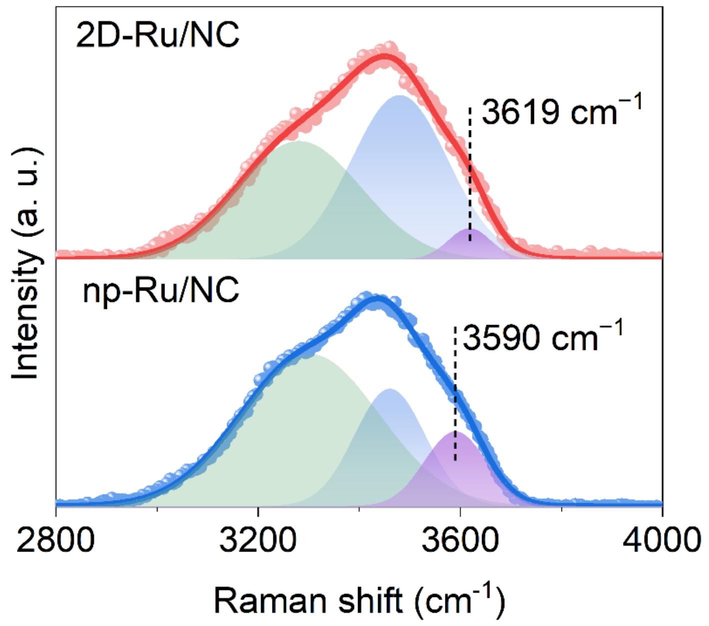

Supplementary Figure 31. *In situ* Raman spectra of interfacial water on 2D-Ru/NC and np-Ru/NC electrode surface at −1.4 V vs. Ag/AgCl. Gaussian fits of three O–H stretching models of 4-HB·H2O, 2-HB·H2O, and $K^+ \cdot H_2O$ are shown in green, blue, and purple, respectively. The vibrational frequency of $K^+ \cdot H_2O$ on 2D-Ru/NC (3618 cm^{-1}) is higher than that of np-Ru/NC (3590 cm^{-1}) and the proportion of $K^+ \cdot H_2O$ in the electric double layer of 2D-Ru/NC (4.8%) is much lower than that of np-Ru/NC (14.9%), suggesting a more pronounced repelling effect that the $K^+ \cdot H_2O$ groups are further away from the catalyst surface and have weaker interaction with the electron-deficient surface of 2D-Ru/NC electrode.

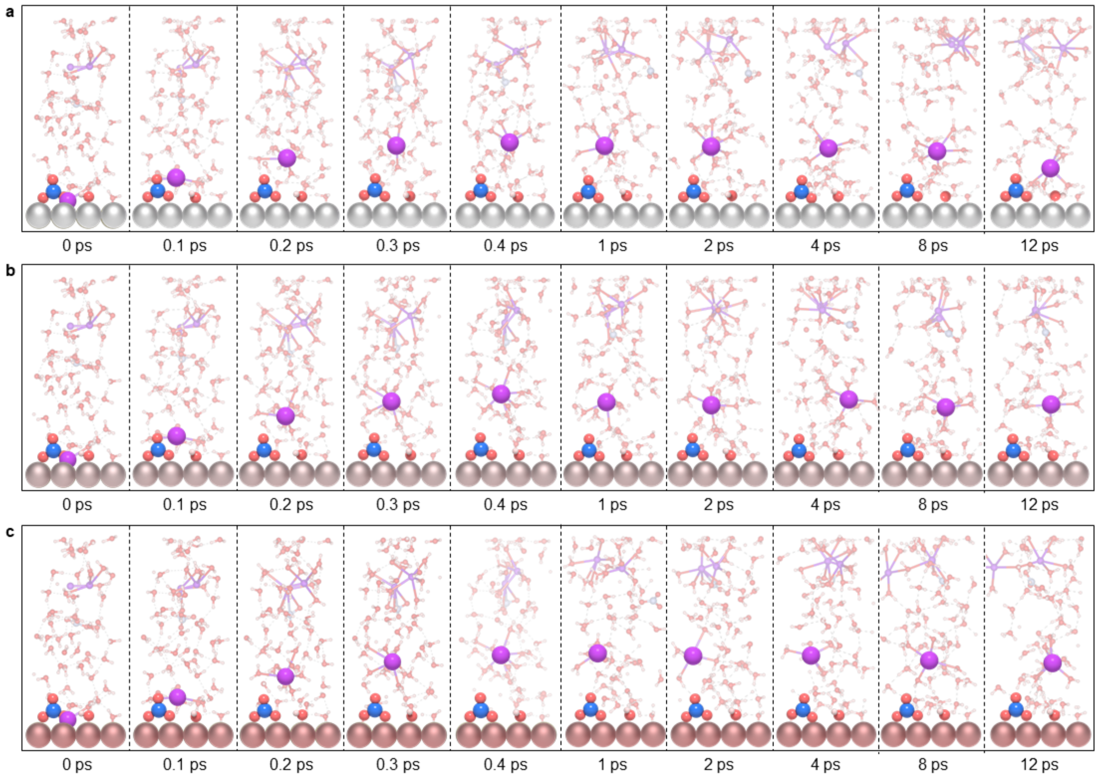

Supplementary Figure 32. a-c Representative snapshots of pre-adsorbed various ions on the Ru (**a**), Ru-0.02 (**b**), and Ru-0.04 (**c**) model surface at different times during AIMD simulation process.

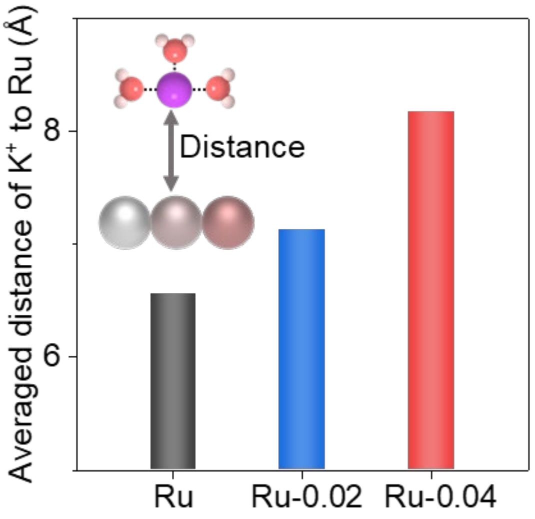

Supplementary Figure 33. Average distance of pre-adsorbed K^+ ion to the surface of neutral Ru, Ru-0.02, and Ru-0.04 models obtained from the last 10 picoseconds of simulations. The average distance of K^+ ion from the Ru surface is 6.6, 7.1, and 8.2 Å for Ru, Ru-0.02, and Ru-0.04 models, respectively. The gradually increased distance matches well with the trend of gradually enhanced electron deficiency of Ru metals, indicating the electron-density dependent repelling effect of hydrated K^+ ions.

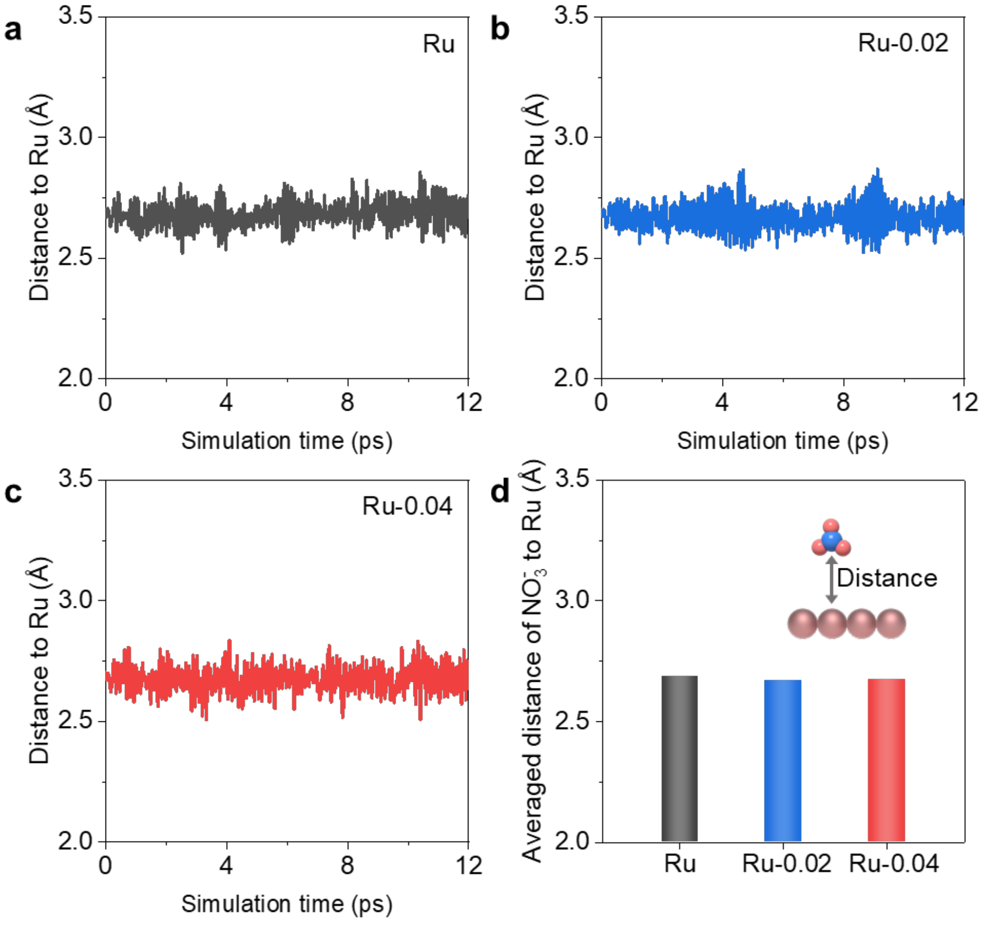

Supplementary Figure 34 a-c Distance of pre-adsorbed NO_3^- ion to the surface of neutral Ru (**a**), Ru-0.02 (**b**), and Ru-0.04 (**c**) models. **d** Average distance of pre-adsorbed NO_3^- ion to the surface of neutral Ru, Ru-0.02, and Ru-0.04 models obtained from the last 10 picoseconds of simulations. The distance is represented by the distance from the N atom in NO_3^- to Ru surface. According to the trajectories and average distances, the NO_3^- ions are not repelled away by the electron-deficient Ru surface but are stably adsorbed on the catalyst surface.

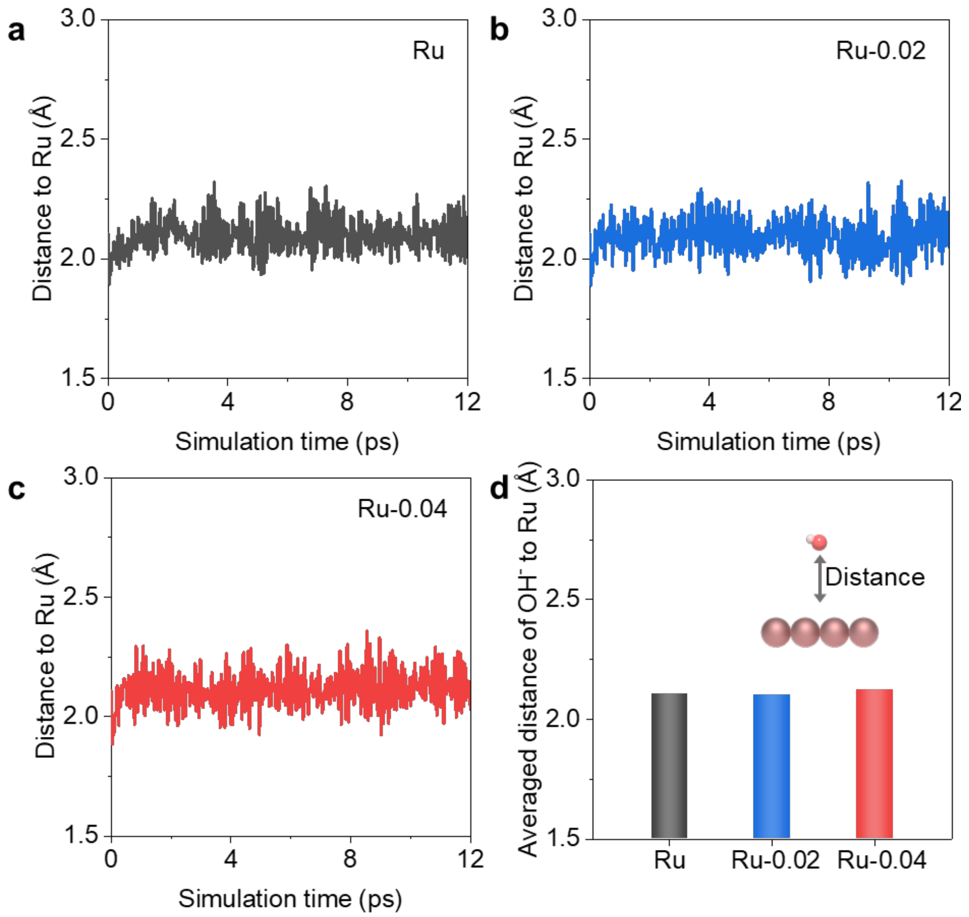

Supplementary Figure 35. a-c Distance of pre-adsorbed OH^- ion to the surface of neutral Ru (**a**), Ru-0.02 (**b**), and Ru-0.04 (**c**) models. **d** Average distance of pre-adsorbed OH^- ion to the surface of neutral Ru, Ru-0.02, and Ru-0.04 models obtained from the last 10 picoseconds of simulations. The distance is represented by the distance from the O atom in OH^- to Ru surface. According to the trajectories and average distances, the OH^- ions are not repelled away by the electron-deficient Ru surface either.

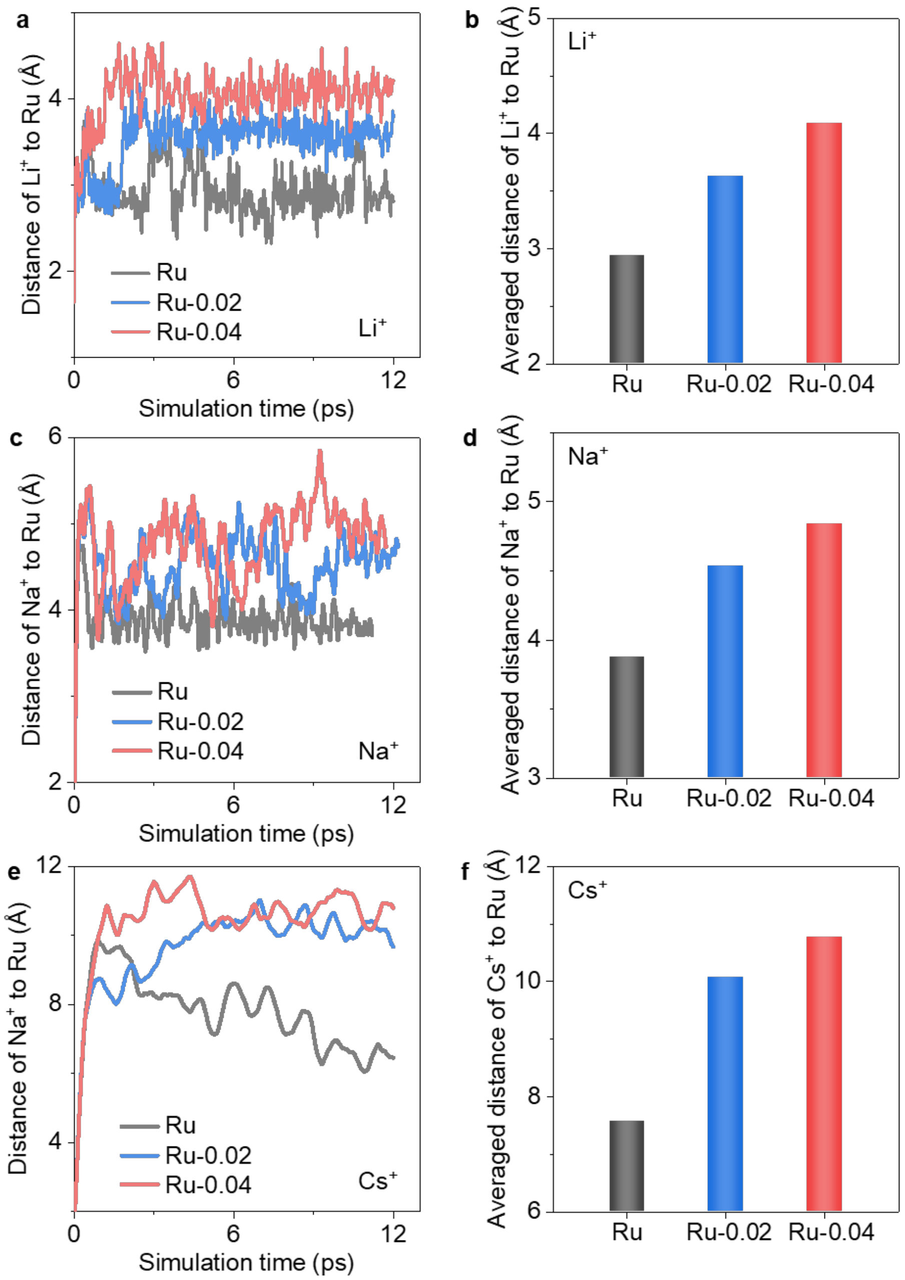

Supplementary Figure 36. a,c,e Distance of pre-adsorbed Li^+ (**a**), Na^+ (**c**), and Cs^+ (**e**) ion to the surface of neutral Ru, Ru-0.02, and Ru-0.04 models. **b,d,e** Average distance of pre-adsorbed Li^+(**b**), Na^+ (**d**), and Cs^+ (**f**) ion to the surface of neutral Ru, Ru-0.02, and Ru-0.04 models obtained from the last 10 picoseconds of simulations. the AIMD simulation results indicate that the unique cation repelling effect of electron-deficient Ru surface is also available for Li^+, Na^+ and Cs^+ ions.

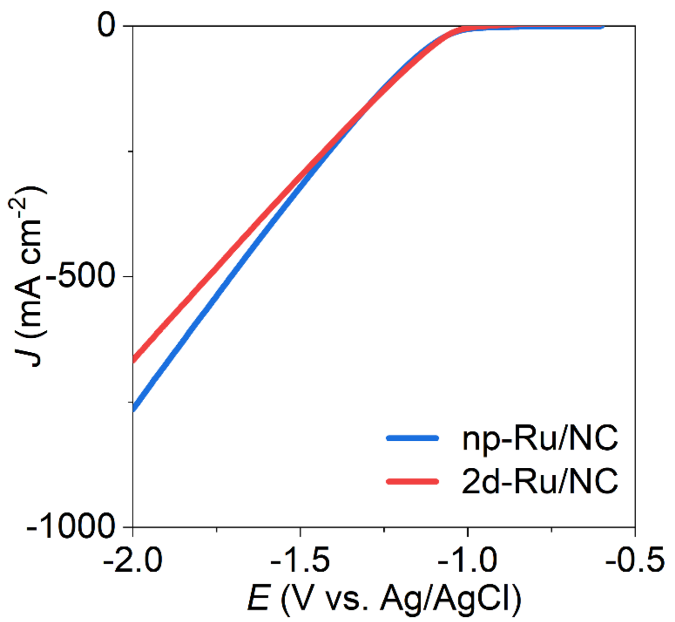

Supplementary Figure 37. LSV curves of 2D-Ru/NC and np-Ru/NC based electrodes in 0.5 M KOH. The relatively higher working potentials for the 2D-Ru/NC based electrode as compared to the np-Ru/NC electrode with the same catalyst loading (1 mg cm^{-2}) directly reveals the slightly depressed HER activity of the 2D Ru metal presumably due to the electron-density dependent K^+ ions repelling effect, well explaining the high and durable FE values over the 2D-Ru/NC based electrode for NO_3RR.

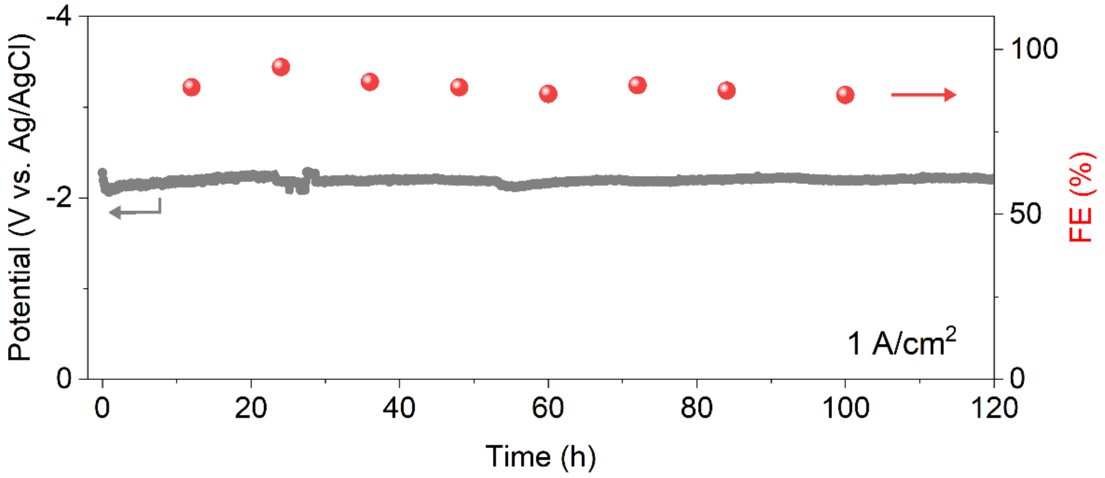

Supplementary Figure 38. Chronopotentiometry curves (gray line) and FE values (red sphere) of NO_3RR over 2D-Ru/NC electrodes at 1 A cm^{-2} in 1 M KNO_3 solution. The total volume of the cathode electrolyte is 1000 mL to avoid a significant decrease in nitrate concentration during long-term test. The well-maintained working voltage during 120-hour ampere level test without obvious Ru leaching (Table S1) again speaks for the good stability of the 2D-Ru/NC electrode even at ampere level.

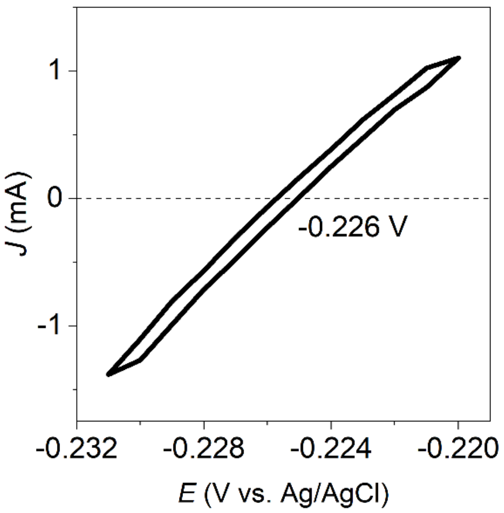

Supplementary Figure 39. Cyclic voltammetry curve for Ag/AgCl electrode calibration in pure H_2-saturated 0.5 M H_2SO_4 solution. Pt meshes are used as both the working electrode and counter electrode. The scan rate is 1 mV s^{-1}.

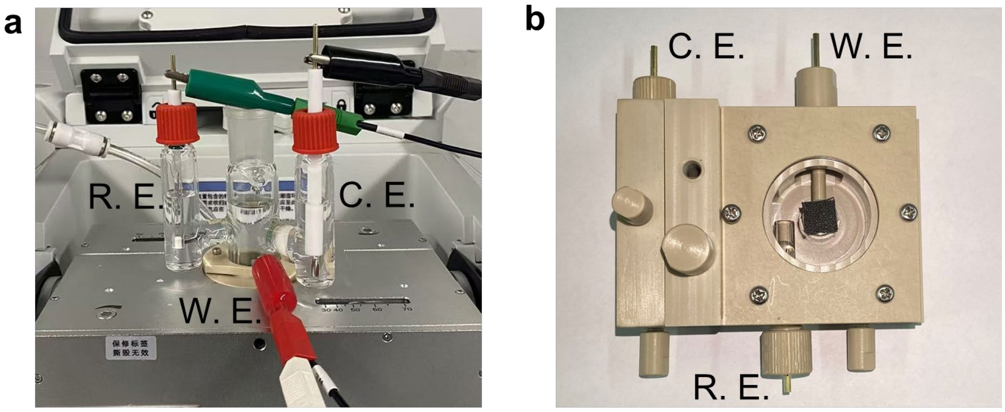

Supplementary Figure 40. a-b Photographs of *in situ* FTIR (**a**) and Raman (**b**) set-up. The working electrode (W. E.), counter electrode (C. E.) and reference electrode (R. E.) are labeled in the photographs.

Table S1-S8

Table S1. Ru contents of different samples obtained by ICP-AES results.

Sample	Ru content by ICP-AES (wt.%)
a-Ru/NC	4.41
2D-Ru/NC	3.82
np-Ru/NC	4.43
np-Ru/C	4.82
Electrolyte after 2-hour test at −1.1 V vs. RHE	-*
Electrolyte after 120-hour ampere level test	-*

*: Below the detection limit (0.1 ppm) of the instrument.

Table S2. Structure parameters derived from the EXAFS simulation of 2D-Ru/NC simple with different fitting models

Adsorbates	Path	CN	R (Å)	σ^2 (10^{-3}Å^2)	ΔE (eV)	R-factor
**Graphite carbon**	Ru-C	8.2	2.05	8.8	-7.22	0.012
	Ru-Ru	3.3	2.77	15.5	4.28	
**Pyridine nitrogen**	Ru-N	6.4	2.02	9.2	-6.36	0.006
	Ru-Ru	2.7	2.79	13.2	7.14	
**Pyrrole nitrogen**	Ru-N	6.5	2.02	9.2	-5.79	0.004
	Ru-Ru	2.8	2.79	13.8	6.55	
**O_2**	Ru-O	4.7	1.99	8.2	-6.77	0.004
	Ru-Ru	1.9	2.76	10.4	4.08	
**H_2O**	Ru-O	4.9	1.98	8.7	-8.31	0.01
	Ru-Ru	1.6	2.77	8.9	5.12	
**Hydroxyl**	Ru-O	4.8	1.99	8.4	-6.98	0.005
	Ru-Ru	1.8	2.77	10.0	4.78	
**Cl**	Ru-Cl	5.92	2.1	14.7	-27.19	0.007
	Ru-Ru	2.2	2.74	11.5	-0.26	

CN is the coordination number, R is the interatomic distance, σ^2 is the Debye−Waller disorder factor, ΔE is the absorption edge offset. All the fittings were conducted with same S_0^2 value. When using Cl as adsorbate for curve fitting, the ΔE value (−27.19 eV) is unacceptable, proving that Cl is not suitable as counter ions on 2D Ru nanosheet for curve fitting. According to the fitting results using graphite carbon, pyridine nitrogen, pyrrole nitrogen, O_2, H_2O, and hydroxyl, the average coordination number of Ru–Ru bond is calculated as 2.4 and average bond distance of Ru–Ru bond is calculated as 2.77 Å in the 2D-Ru/NC sample.

Table S3. Structure parameters derived from the EXAFS simulation of various samples.

Sample	Path	CN	R (Å)	σ^2 (10^{-3}Å^2)	ΔE (eV)	R-factor
Ru foil	Ru-Ru	12	2.68	3.64	2.76	0.010
np-Ru/NC	Ru-Ru	10.3	2.68	4.54	-8.04	0.020
RuO_2	Ru-O	6	1.98	3.85	0.12	0.018
	Ru-O-Ru	8	3.20	7.04	0.26	

CN is the coordination number, R is the interatomic distance, σ^2 is the Debye−Waller disorder factor, ΔE is the absorption edge offset.

Table S4. Gibbs free energy change of each step in the proposed mechanism of NO_3RR on Ru and Ru-0.04 model.

**Reaction path**	**Ru model (eV)**	**Ru-0.04 model (eV)**
$NO_3^- \rightarrow *NO_3$	-2.88	-3.01
$*NO_3 \rightarrow *NO_2$	-1.95	-1.92
$*NO_2 \rightarrow *NO$	-2.09	-2.13
$*NO \rightarrow *NOH$	0.91	0.79
$*NOH \rightarrow *NHOH$	-0.57	-0.50
$*NHOH \rightarrow *NH_2OH$	-0.27	-0.31
$*NH_2OH \rightarrow *NH_2$	-1.64	-1.62
$*NH_2 \rightarrow *NH_3$	-0.68	-0.78

Table S5. NO_3RR performance of the 2D-Ru/NC and state-of-art electrocatalysts in neutral condition.

Sample	Electrolyte	J (mA/cm^{-2})	NH_3 yield (mg cm^{-2} h^{-1})	FE (%)	Ref.
**2D-Ru/NC**	**1 M KNO_3**	**~700**	**56.3**	**> 99**	**This work**
		**1000**	**74.8**	**94**	
Ni_{35}/NC-sd	0.3 M NaNO3 + 0.5 M $NsSO_4$	~60	5.1	> 99	Ref. 3
Ru-CuNW	0.0323 M of $NaNO_3$	100	7.3	92	Ref. 4
Ru-POC	1 M KNO_3	~140	11.2	96	Ref. 5
Fe@Cu_1FeO_x	1000 ppm NO_3^--N + 0.1 M K2SO4	-	1.98	95.4	Ref. 6
Fe-HESA NCs	100 mM $NaNO_3$ and 0.5 M Na_2SO_4	~53	4.07	93.4	Ref. 7
CoP/Zn-ZIF	0.1 M NO_3^-+0.5 M Na_2SO_4	75	15.13	97.2	Ref. 8
a-RuO2	200 ppm $NaNO_3$ + 0.5 M Na_2SO_4	~50	1.97	97.5	Ref. 9
$LaCoO_3$	1 M Na_2SO_4 and 0.5 M KNO_3	~140	10.15	91.5	Ref. 10
MAT-CoNi/CF	0.1 M KNO_3 + 0.5 M K_2SO_4	~360	25	98.5	Ref. 11
Pd/TiO_2	0.25 M $LiNO_3$ + 1 M LiCl	~20	1.12	92.1	Ref. 12
Fe/Ni_2P	0.05 M KNO_3 + 0.2 M K_2SO_4	~60	4.17	94.3	Ref. 13
Cu@hNCNC	50 mM KNO_3 + 0.5 M Na_2SO_4	~170	8.16	> 99	Ref. 14
I_1Cu_4	0.5 M K_2SO_4 + 100 mg/L NO_3^-	~90	4.36	98.5	Ref. 15

Table S6. NO_3RR performance of the 2D-Ru/NC and state-of-art Ru-based electrocatalysts

Sample	Electrolyte	J (mA/cm^{-2})	NH_3 yield (mg cm^{-2} h^{-1})	FE (%)	Ref.
**2D-Ru/NC**	**1 M KNO_3**	**~700**	**56.3**	**> 99**	**This work**
		**1000**	**74.8**	**94**	
Ru-CuNW	0.0323 M of $NaNO_3$	100	7.3	92	Ref. 4
Ru/Cu_2O	1 M KNO_3 + 1 M KOH	467	37.1	> 99	Ref. 16
G-RuCo	32.3 mM KNO_3 + 1 M KOH	585	51.5	97.8	Ref. 17
Ru-POC	1 M KNO_3	~140	11.2	96	Ref. 5
Ru_1Cu_{10}/rGO	0.1 M KNO_3 + 1 M KOH	89	6.5	98	Ref. 18
a-RuO2	200 ppm $NaNO_3$ + 0.5 M Na_2SO_4	~50	1.97	97.5	Ref. 9
$Ru_{15}Co_{85}$ HNDs	0.1 M KNO_3 + 0.1 M KOH	-	20.9	96.8	Ref. 19
Ru/β-Co(OH)2	0.1 M KNO_3 + 1 M KOH	500	39.1	98.8	Ref. 20
Ru-Fe_2O_3	0.1 M $NaNO_3$ + 0.5 M Na_2SO_4	~130	5.6	72.7	Ref. 21
RuNi-MOF	200 ppm $NaNO_3$ + 0.1 M Na_2SO_4	30.6	1.4	63.6%	Ref. 22
Ru-Co CHNWs	1 M $NaNO_3$ + 1 M NaOH	~100	8.21	> 99	Ref. 23
RuFe NFs	0.1 M $NaNO_3$ + 0.5 M Na_2SO_4	~10	0.27	92.9	Ref. 24
$RuIn_3$/C	0.01 M KNO_3 + 0.1 M KOH	~23	1.11	97.6	Ref. 25
Ru NCs/TiO_2 NTs	100 ppm KNO_3 + 0.05 M Na_2SO_4	~12	0.6	90	Ref. 26
Ru-ST-12	1 M KNO_3 + 1 M KOH	125	19.9	> 99	Ref. 27

Table S7. The proportions of the three types of interfacial water on 2D-Ru/NC and np-Ru/NC surface at different potentials.

**Potential (V vs. Ag/AgCl)**	**2D-Ru/NC**			**np-Ru/NC**		
	4-HB-H_2O	2-HB-H_2O	$K^+ \cdot H_2O$	4-HB-H_2O	2-HB-H_2O	$K^+ \cdot H_2O$
0.1	32.0%	64.4%	3.7%	42.3%	43.7%	14.0%
−0.2	31.4%	64.9%	3.7%	43.6%	42.3%	14.1%
−0.5	35.3%	60.9%	3.8%	45.1%	40.4%	14.5%
−0.8	33.3%	62.6%	4.1%	46.6%	39.3%	14.2%
−1.1	44.7%	50.8%	4.6%	50.0%	35.7%	14.3%
−1.4	51.5%	43.7%	4.8%	55.7%	29.4%	14.9%

Table S8. NO_3RR performance under ampere-lever current density over 2D-Ru/NC electrode and other reported Ru-based electrocatalysts

Sample	Electrolyte	NH_3 yield (mg mg_{cat}^{-1} h^{-1})	FE (%)	c(NH_3) (mol/L)	Ref.
**2D-Ru/NC**	**1 M KNO_3**	**74.8**	**94**	**0.26**	**This work**
G-RuCo	32.3 mM KNO_3 + 1 M KOH	11.8	93	0.032	Ref. 17
$Ru_{15}Co_{85}$ HNDs	0.1 M KNO_3 + 0.1 M KOH	27.1	82	0.002	Ref. 19

c(NH_3) is the ammonia concentration of the obtained solutions without any concentration and purification process.

References

1 Song, F. *et al.* Free-standing graphene by scanning transmission electron microscopy. *Ultramicroscopy* **110**, 1460-1464 (2010).

2 Bals, S., Kabius, B., Haider, M., Radmilovic, V. & Kisielowski, C. Annular dark field imaging in a TEM. *Solid State Commun.* **130**, 675-680 (2004).

3 Gao, P. *et al.* Schottky barrier-induced surface electric field boosts universal reduction of NO_x^- in water to ammonia. *Angew. Chem. Int. Ed.* **60**, 20711-20716 (2021).

4 Chen, F. Y. *et al.* Electrochemical nitrate reduction to ammonia with cation shuttling in a solid electrolyte reactor. *Nature Catal.*, **7**, 1032-1043 (2024).

5 Zhai, G. Y. *et al.* Accelerating the activation of NO_x^- on Ru nanoparticles for ammonia production by tuning their electron deficiency. *CCS Chem.* **4**, 3455-3462 (2022).

6 Zhou, B. *et al.* Cu_1-Fe dual sites for superior neutral ammonia electrosynthesis from nitrate. *Angew. Chem. Int. Ed.* **63**, e202406046 (2024).

7 Tang, S. et al. General synthesis of high-entropy single-atom nanocages for electrosynthesis of ammonia from nitrate. *Nat. Commun.* **15**, 6932 (2024).

8 Zhang, C. et al. Enabling logistics automation in nanofactory: cobalt phosphide embedded metal–organic frameworks for efficient electrocatalytic nitrate reduction to ammonia. *Adv. Mater.* **36**, 2313844 (2024).

9 Wang, Y. et al. Structurally disordered RuO_2 nanosheets with rich oxygen vacancies for enhanced nitrate electroreduction to ammonia. *Angew. Chem. Int. Ed.* **134**, e202202604 (2022).

10 Zheng, H. et al. Perovskites with enriched oxygen vacancies as a family of electrocatalysts for efficient nitrate reduction to ammonia. *Small* **19**, 2205625 (2023).

11 Wei, Y. et al. Electrocatalytic nitrate reduction on metallic CoNi-terminated catalyst with industrial-level current density in neutral medium. *Adv. Mater.* **36**, 2404774 (2024).

12 Guo, Y. et al. Pd doping-weakened intermediate adsorption to promote electrocatalytic nitrate reduction on TiO_2 nanoarrays for ammonia production and energy supply with zinc–nitrate batteries. *Energy Eeviron. Sci.* **14**, 3938-3944 (2021).

13 Zhang, R. et al. Efficient ammonia electrosynthesis and energy conversion through a Zn-nitrate battery by iron doping engineered nickel phosphide catalyst. *Adv. Energy Mater.* **12**, 2103872 (2022).

14 Shen, Z. et al. Self-enhanced localized alkalinity at the encapsulated Cu catalyst for superb electrocatalytic nitrate/nitrite reduction to NH_3 in neutral electrolyte. *Sci. Adv.* **10**, eadm9325 (2024).

15 Zhou, B. et al. Reversed I_1Cu_4 single-atom sites for superior neutral ammonia electrosynthesis with nitrate. *Proc. Natl. Acad. Sci. USA* **121**, e2405236121 (2024).

16 Hu, Q. *et al.* Ammonia electrosynthesis from nitrate using a ruthenium–copper cocatalyst system: a full concentration range study. *J. Am. Chem. Soc.* **146**, 668-676 (2023).
17 Chen, X., Cheng, Y., Zhang, B., Zhou, J. & He, S. Gradient-concentration RuCo electrocatalyst for efficient and stable electroreduction of nitrate into ammonia. *Nat. Commun.* **15**, 6278 (2024).
18 Gao, W. *et al.* Alloying of Cu with Ru enabling the relay catalysis for reduction of nitrate to ammonia. *Adv. Mater.* **35**, 2202952 (2023).
19 Han, S. *et al.* Ultralow overpotential nitrate reduction to ammonia via a three-step relay mechanism. *Nat. Catal.* **6**, 402-414 (2023).
20 Zhu, W. *et al.* Weakened d–p orbital hybridization in in situ reconstructed Ru/β-$Co(OH)_2$ heterointerfaces for accelerated ammonia electrosynthesis from nitrates. *Energy Environ. Sci.* **16**, 2483-2493 (2023).
21 Luo, S. *et al.* Ruthenium-induced hydrolysis effect on Fe_2O_3 nanoarrays for high-performance electrochemical nitrate reduction to ammonia. *Appl. Catal. B* **351**, 123967 (2024).
22 Qin, J. *et al.* Achieving high selectivity for nitrate electrochemical reduction to ammonia over MOF-supported Ru_xO_y clusters. *J. Mater. Chem. A* **10**, 3963-3969 (2022).
23 Cao, H. *et al.* Constructing Ru single-atomic sites through potential-induced self-reconstruction to accelerate electrocatalytic nitrate reduction for ammonia production. *Chem. Eng. J.* **490**, 151883 (2024).
24 Wang, Y. *et al.* Atomic coordination environment engineering of bimetallic alloy nanostructures for efficient ammonia electrosynthesis from nitrate. *Proc. Natl. Acad. Sci. USA* **120**, e2306461120 (2023).
25 Huang, Y. *et al.* Pulsed electroreduction of low-concentration nitrate to ammonia. *Nat. Commun.* **14**, 7368 (2023).
26 Qiu, W. *et al.* Size-defined Ru nanoclusters supported by TiO_2 nanotubes enable low-concentration nitrate electroreduction to ammonia with suppressed hydrogen evolution. *Small* **19**, 2300437 (2023).
27 Li, J. *et al.* Efficient ammonia electrosynthesis from nitrate on strained ruthenium nanoclusters. *J. Am. Chem. Soc.* **142**, 7036-7046 (2020).
