# Supplementary material for: Ampere-level reduction of pure nitrate by electron-deficient Ru with K+ ions repelling effect
Source: Nat Commun. 2024 Dec 30;15:10877. doi: 10.1038/s41467-024-55230-w (PMC11685401; doi:10.1038/s41467-024-55230-w)
Supplement: Supplementary file 2 — Description Of Additional Supplementary File [file 41467_2024_55230_MOESM2_ESM.pdf]

Description of Additional supplementary files

Supplementary Data 1

Atomic coordinate information for the DFT calculated 2D-Ru/NC model

Supplementary Data 2

Atomic coordinate information for the DFT calculated np-Ru/NC model

Supplementary Data 3

Atomic coordinate information of the initial configuration for Ru-0.04 model for AIMD simulation

Supplementary Data 4

Atomic coordinate information of the final configuration for Ru-0.04 model for AIMD simulation
