# Supplementary material for: Ampere-level reduction of pure nitrate by electron-deficient Ru with K+ ions repelling effect
Source: Nat Commun. 2024 Dec 30;15:10877. doi: 10.1038/s41467-024-55230-w (PMC11685401; doi:10.1038/s41467-024-55230-w)
Supplement: Supplementary file 3 — Supplementary Data 1 [file 41467_2024_55230_MOESM3_ESM.docx]

2D-Ru

1.0

17.2199993134 0.0000000000 0.0000000000

-8.6099996567 14.9129568585 0.0000000000

0.0000000000 0.0000000000 20.0000000000

C N Ru

91 6 20

Direct

0.000000000 0.000000000 0.085000001

0.095239997 0.047619998 0.085000001

0.142859995 0.000000000 0.085000001

0.238100007 0.047619998 0.085000001

0.285710007 0.000000000 0.085000001

0.380950004 0.047619998 0.085000001

0.428570002 0.000000000 0.085000001

0.523810029 0.047619998 0.085000001

0.571430027 0.000000000 0.085000001

0.666670024 0.047619998 0.085000001

0.714290023 0.000000000 0.085000001

0.809520006 0.047619998 0.085000001

0.857140005 0.000000000 0.085000001

0.952380002 0.047619998 0.085000001

0.000000000 0.142859995 0.085000001

0.095239997 0.190479994 0.085000001

0.142859995 0.142859995 0.085000001

0.238100007 0.190479994 0.085000001

0.380950004 0.190479994 0.085000001

0.428570002 0.142859995 0.085000001

0.523810029 0.190479994 0.085000001

0.571430027 0.142859995 0.085000001

0.666670024 0.190479994 0.085000001

0.714290023 0.142859995 0.085000001

0.857140005 0.142859995 0.085000001

0.952380002 0.190479994 0.085000001

0.000000000 0.285710007 0.085000001

0.095239997 0.333330005 0.085000001

0.142859995 0.285710007 0.085000001

0.238100007 0.333330005 0.085000001

0.285710007 0.285710007 0.085000001

0.380950004 0.333330005 0.085000001

0.428570002 0.285710007 0.085000001

0.523810029 0.333330005 0.085000001

0.571430027 0.285710007 0.085000001

0.666670024 0.333330005 0.085000001

0.714290023 0.285710007 0.085000001

0.809520006 0.333330005 0.085000001

0.857140005 0.285710007 0.085000001

0.952380002 0.333330005 0.085000001

0.000000000 0.428570002 0.085000001

0.095239997 0.476190001 0.085000001

0.142859995 0.428570002 0.085000001

0.238100007 0.476190001 0.085000001

0.285710007 0.428570002 0.085000001

0.380950004 0.476190001 0.085000001

0.428570002 0.428570002 0.085000001

0.666670024 0.476190001 0.085000001

0.714290023 0.428570002 0.085000001

0.809520006 0.476190001 0.085000001

0.857140005 0.428570002 0.085000001

0.952380002 0.476190001 0.085000001

0.000000000 0.571430027 0.085000001

0.095239997 0.619050026 0.085000001

0.142859995 0.571430027 0.085000001

0.238100007 0.619050026 0.085000001

0.285710007 0.571430027 0.085000001

0.428570002 0.571430027 0.085000001

0.523810029 0.619050026 0.085000001

0.571430027 0.571430027 0.085000001

0.666670024 0.619050026 0.085000001

0.714290023 0.571430027 0.085000001

0.809520006 0.619050026 0.085000001

0.857140005 0.571430027 0.085000001

0.952380002 0.619050026 0.085000001

0.000000000 0.714290023 0.085000001

0.095239997 0.761900008 0.085000001

0.142859995 0.714290023 0.085000001

0.285710007 0.714290023 0.085000001

0.380950004 0.761900008 0.085000001

0.428570002 0.714290023 0.085000001

0.523810029 0.761900008 0.085000001

0.571430027 0.714290023 0.085000001

0.666670024 0.761900008 0.085000001

0.714290023 0.714290023 0.085000001

0.809520006 0.761900008 0.085000001

0.857140005 0.714290023 0.085000001

0.952380002 0.761900008 0.085000001

0.000000000 0.857140005 0.085000001

0.095239997 0.904760003 0.085000001

0.142859995 0.857140005 0.085000001

0.238100007 0.904760003 0.085000001

0.285710007 0.857140005 0.085000001

0.380950004 0.904760003 0.085000001

0.428570002 0.857140005 0.085000001

0.523810029 0.904760003 0.085000001

0.571430027 0.857140005 0.085000001

0.666670024 0.904760003 0.085000001

0.714290023 0.857140005 0.085000001

0.809520006 0.904760003 0.085000001

0.952380002 0.904760003 0.085000001

0.285710007 0.142859995 0.085000001

0.809520006 0.190479994 0.085000001

0.571430027 0.428570002 0.085000001

0.380950004 0.619050026 0.085000001

0.238100007 0.761900008 0.085000001

0.857140005 0.857140005 0.085000001

0.297317386 0.165937036 0.222000003

0.418056607 0.138601139 0.222000003

0.548787117 0.120841198 0.222000003

0.695015669 0.161982253 0.222000003

0.263577908 0.279647321 0.222000003

0.458049983 0.294090599 0.222000003

0.590196490 0.274861723 0.222000003

0.737949193 0.311669827 0.222000003

0.301269501 0.464230895 0.222000003

0.406152904 0.400491238 0.222000003

0.587659419 0.435013562 0.222000003

0.732726038 0.452131778 0.222000003

0.284115076 0.582747936 0.222000003

0.462009132 0.561592758 0.222000003

0.606917262 0.573954046 0.222000003

0.783461690 0.602265000 0.222000003

0.341989547 0.733283877 0.222000003

0.458185315 0.703216255 0.222000003

0.599547088 0.717793405 0.222000003

0.748403907 0.715059996 0.222000003
