# Supplementary material for: Ampere-level reduction of pure nitrate by electron-deficient Ru with K+ ions repelling effect
Source: Nat Commun. 2024 Dec 30;15:10877. doi: 10.1038/s41467-024-55230-w (PMC11685401; doi:10.1038/s41467-024-55230-w)
Supplement: Supplementary file 4 — Supplementary Data 2 [file 41467_2024_55230_MOESM4_ESM.docx]

np-Ru

1.0

17.2199993134 0.0000000000 0.0000000000

-8.6099996567 14.9129568585 0.0000000000

0.0000000000 0.0000000000 20.0000000000

C N Ru

91 6 20

Direct

0.000000000 0.000000000 0.085000001

0.095239997 0.047619998 0.085000001

0.142859995 0.000000000 0.085000001

0.238100007 0.047619998 0.085000001

0.285710007 0.000000000 0.085000001

0.380950004 0.047619998 0.085000001

0.428570002 0.000000000 0.085000001

0.523810029 0.047619998 0.085000001

0.571430027 0.000000000 0.085000001

0.666670024 0.047619998 0.085000001

0.714290023 0.000000000 0.085000001

0.809520006 0.047619998 0.085000001

0.857140005 0.000000000 0.085000001

0.952380002 0.047619998 0.085000001

0.000000000 0.142859995 0.085000001

0.095239997 0.190479994 0.085000001

0.142859995 0.142859995 0.085000001

0.238100007 0.190479994 0.085000001

0.380950004 0.190479994 0.085000001

0.428570002 0.142859995 0.085000001

0.523810029 0.190479994 0.085000001

0.571430027 0.142859995 0.085000001

0.666670024 0.190479994 0.085000001

0.714290023 0.142859995 0.085000001

0.857140005 0.142859995 0.085000001

0.952380002 0.190479994 0.085000001

0.000000000 0.285710007 0.085000001

0.095239997 0.333330005 0.085000001

0.142859995 0.285710007 0.085000001

0.238100007 0.333330005 0.085000001

0.285710007 0.285710007 0.085000001

0.380950004 0.333330005 0.085000001

0.428570002 0.285710007 0.085000001

0.523810029 0.333330005 0.085000001

0.571430027 0.285710007 0.085000001

0.666670024 0.333330005 0.085000001

0.714290023 0.285710007 0.085000001

0.809520006 0.333330005 0.085000001

0.857140005 0.285710007 0.085000001

0.952380002 0.333330005 0.085000001

0.000000000 0.428570002 0.085000001

0.095239997 0.476190001 0.085000001

0.142859995 0.428570002 0.085000001

0.238100007 0.476190001 0.085000001

0.285710007 0.428570002 0.085000001

0.380950004 0.476190001 0.085000001

0.428570002 0.428570002 0.085000001

0.666670024 0.476190001 0.085000001

0.714290023 0.428570002 0.085000001

0.809520006 0.476190001 0.085000001

0.857140005 0.428570002 0.085000001

0.952380002 0.476190001 0.085000001

0.000000000 0.571430027 0.085000001

0.095239997 0.619050026 0.085000001

0.142859995 0.571430027 0.085000001

0.238100007 0.619050026 0.085000001

0.285710007 0.571430027 0.085000001

0.428570002 0.571430027 0.085000001

0.523810029 0.619050026 0.085000001

0.571430027 0.571430027 0.085000001

0.666670024 0.619050026 0.085000001

0.714290023 0.571430027 0.085000001

0.809520006 0.619050026 0.085000001

0.857140005 0.571430027 0.085000001

0.952380002 0.619050026 0.085000001

0.000000000 0.714290023 0.085000001

0.095239997 0.761900008 0.085000001

0.142859995 0.714290023 0.085000001

0.285710007 0.714290023 0.085000001

0.380950004 0.761900008 0.085000001

0.428570002 0.714290023 0.085000001

0.523810029 0.761900008 0.085000001

0.571430027 0.714290023 0.085000001

0.666670024 0.761900008 0.085000001

0.714290023 0.714290023 0.085000001

0.809520006 0.761900008 0.085000001

0.857140005 0.714290023 0.085000001

0.952380002 0.761900008 0.085000001

0.000000000 0.857140005 0.085000001

0.095239997 0.904760003 0.085000001

0.142859995 0.857140005 0.085000001

0.238100007 0.904760003 0.085000001

0.285710007 0.857140005 0.085000001

0.380950004 0.904760003 0.085000001

0.428570002 0.857140005 0.085000001

0.523810029 0.904760003 0.085000001

0.571430027 0.857140005 0.085000001

0.666670024 0.904760003 0.085000001

0.714290023 0.857140005 0.085000001

0.809520006 0.904760003 0.085000001

0.952380002 0.904760003 0.085000001

0.285710007 0.142859995 0.085000001

0.809520006 0.190479994 0.085000001

0.571430027 0.428570002 0.085000001

0.380950004 0.619050026 0.085000001

0.238100007 0.761900008 0.085000001

0.857140005 0.857140005 0.085000001

0.430100292 0.417856723 0.222000003

0.586752653 0.428054154 0.222000003

0.426669002 0.559673488 0.222000003

0.575628281 0.568609953 0.222000003

0.725927114 0.571648061 0.222000003

0.571871102 0.713365316 0.222000003

0.719432712 0.718859375 0.222000003

0.381248862 0.468537003 0.329040021

0.433131665 0.418467611 0.436080009

0.525648177 0.465485305 0.329040021

0.588065445 0.425933212 0.436080009

0.669425130 0.465017706 0.329040021

0.425617963 0.556093276 0.436080009

0.522996128 0.618034124 0.329040021

0.575476527 0.565459013 0.436080009

0.678802729 0.618751228 0.329040021

0.723334253 0.571469188 0.436080009

0.571035147 0.708978713 0.436080009

0.674382031 0.761211038 0.329040021

0.716327667 0.716618359 0.436080009
