# Supplementary material for: Ampere-level reduction of pure nitrate by electron-deficient Ru with K+ ions repelling effect
Source: Nat Commun. 2024 Dec 30;15:10877. doi: 10.1038/s41467-024-55230-w (PMC11685401; doi:10.1038/s41467-024-55230-w)
Supplement: Supplementary file 5 — Supplementary Data 3 [file 41467_2024_55230_MOESM5_ESM.docx]

POSCAR file written by OVITO Basic 3.7.6

1.0

10.8232002258 0.0000000000 0.0000000000

-5.4116001129 9.3731663458 0.0000000000

0.0000000000 0.0000000000 23.3885002136

Ru O N K H

16 61 2 3 109

Direct

0.083329998 0.166669995 0.057290003

0.333330005 0.166669995 0.057290003

0.583329976 0.166669995 0.057290003

0.833329976 0.166669995 0.057290003

0.083330013 0.416669995 0.057290003

0.333330005 0.416669995 0.057290003

0.583329976 0.416669995 0.057290003

0.833329976 0.416669995 0.057290003

0.083330013 0.666670024 0.057290003

0.333330005 0.666670024 0.057290003

0.583329976 0.666670024 0.057290003

0.833329976 0.666670024 0.057290003

0.083329976 0.916670024 0.057290003

0.333330005 0.916670024 0.057290003

0.583329976 0.916670024 0.057290003

0.833329976 0.916670024 0.057290003

0.331380010 0.217790008 0.815959990

0.964950085 0.344909996 0.805450022

0.044909999 0.425529987 0.242330000

0.603510022 0.596840024 0.911520004

0.561049998 0.247569993 0.716400027

0.451270014 0.085239999 0.213520005

0.276030004 0.205410004 0.619809985

0.837880015 0.912649930 0.799889982

0.538699985 0.493909985 0.802309990

0.339280009 0.646889985 0.609740019

0.182119995 0.687020004 0.194530010

0.788709998 0.668299973 0.215619996

0.677770019 0.640749991 0.322279990

0.439379990 0.519869983 0.698430002

0.861649990 0.235730007 0.916670024

0.872990012 0.694019973 0.832779944

0.381749988 0.047230002 0.512030005

0.027269987 0.620410025 0.503139973

0.908299983 0.377929986 0.563319981

0.796199977 0.140799999 0.196830004

0.392170042 0.955810070 0.954779983

0.288520008 0.634050012 0.916779935

0.334129989 0.901250005 0.409660012

0.858799994 0.878310025 0.971570075

0.566020012 0.415950000 0.604290009

0.521009982 0.008070000 0.630050004

0.579699993 0.456950009 0.449180007

0.361699998 0.332219988 0.380089998

0.755339980 0.825100005 0.410180032

0.486919999 0.760270000 0.435290009

0.801190019 0.762070000 0.701139987

0.634599984 0.267479986 0.522899985

0.878359973 0.873260021 0.141240001

0.320609987 0.437720001 0.965860009

0.695190012 0.077299997 0.441359997

0.516700029 0.775810003 0.821539998

0.050739985 0.782130003 0.277819991

0.916059971 0.794990003 0.492449999

0.185810000 0.313679993 0.461740017

0.516420007 0.972400010 0.753499985

0.589320004 0.848950028 0.557810009

0.807739973 0.446449995 0.187810004

0.744239986 0.710219979 0.582570016

0.592429996 0.674430013 0.146899998

0.184220001 0.270760000 0.722310007

0.871829987 0.143110007 0.698390007

0.147819996 0.346650004 0.577520013

0.608659983 0.300920010 0.923669994

0.963039994 0.150529996 0.379139990

0.133599997 0.807650030 0.897049963

0.694239974 0.917500019 0.269959986

0.462370008 0.842249990 0.205090016

0.328240007 0.067989998 0.323379993

0.142590001 0.072049998 0.516839981

0.107580014 0.674120009 0.372889996

0.324019998 0.431950003 0.148049995

0.137799993 0.194830000 0.142169997

0.252519995 0.278860003 0.223010004

0.305860013 0.917500019 0.560859978

0.297080010 0.738520026 0.587220013

0.081739999 0.740540028 0.594380021

0.217680007 0.793749988 0.578740001

0.242860004 0.304769993 0.172380000

0.814130008 0.469550014 0.754160047

0.217160001 0.676530004 0.750280023

0.596199989 0.243399993 0.127470002

0.286410004 0.232660010 0.781210005

0.414050013 0.314159989 0.811990023

0.896269977 0.375860006 0.789669991

0.931070030 0.316529989 0.846469939

0.069410004 0.353769988 0.230110005

0.097810015 0.517979980 0.220899999

0.564769983 0.622520030 0.945880055

0.580190003 0.501079977 0.924600005

0.577589989 0.291429996 0.679489970

0.492850006 0.272839993 0.733640015

0.462269962 0.999269962 0.204400003

0.471150011 0.140310004 0.178519994

0.229780003 0.128209978 0.590579987

0.373369992 0.275389999 0.606630027

0.743380010 0.892440021 0.814499974

0.813759923 0.863239944 0.762480021

0.545239985 0.525449991 0.761530042

0.560880005 0.572260022 0.827889919

0.266449988 0.545970023 0.601509988

0.393249989 0.638220012 0.640659988

0.161400005 0.697749972 0.154400006

0.286529988 0.724210024 0.200059980

0.787410021 0.569999993 0.202690020

0.756190002 0.658659995 0.255239993

0.717429996 0.712130010 0.351109982

0.677250028 0.558939993 0.341109991

0.479420006 0.475109994 0.673529983

0.346190006 0.436170012 0.709819913

0.922559977 0.287200004 0.949570060

0.781440020 0.253349990 0.918560028

0.803290009 0.638209999 0.862930000

0.854330003 0.775219977 0.831239939

0.487179965 0.113450006 0.518639982

0.371659994 0.002550000 0.473710001

0.111319989 0.694130003 0.528460026

0.075399987 0.608110011 0.470510006

0.954559982 0.460229993 0.538519979

0.965879977 0.337720007 0.575670004

0.732479990 0.093000002 0.164660022

0.863929987 0.237830013 0.187979996

0.450470001 0.909399986 0.950789928

0.305080026 0.899550080 0.929939985

0.213860020 0.561699986 0.890709996

0.243540004 0.643069983 0.952069998

0.358919978 0.961849928 0.374650002

0.238470018 0.813510001 0.393580019

0.849950016 0.962840021 0.980240047

0.767870009 0.795459986 0.957740009

0.624670029 0.512080014 0.587100029

0.590910017 0.351509988 0.580879986

0.602419972 0.107129999 0.630309999

0.556289971 0.966910064 0.601040006

0.569739997 0.503629982 0.483370036

0.496039987 0.420590013 0.422650009

0.369590014 0.277029991 0.348010004

0.371190012 0.416810006 0.362480044

0.810249984 0.810220003 0.441619962

0.818960011 0.921130002 0.393669993

0.579900026 0.795480013 0.417169988

0.448009998 0.827269971 0.431899995

0.826049984 0.697300017 0.718949914

0.785220027 0.737519979 0.660130024

0.621720016 0.310220003 0.486059964

0.741970003 0.320719987 0.533819973

0.866360009 0.802110016 0.172070011

0.923690021 0.972409964 0.150120005

0.242860004 0.341769993 0.958130002

0.300949991 0.511669993 0.946730018

0.753859997 0.061600000 0.468959957

0.679019988 0.145689994 0.463440001

0.476449996 0.780969977 0.858210027

0.507120013 0.842040002 0.795589983

0.969219983 0.718490005 0.253809988

0.134500012 0.809790015 0.252640009

0.997219980 0.897049963 0.497409999

0.958440006 0.730440021 0.491549999

0.104680009 0.295170009 0.436030000

0.246290013 0.296400011 0.434879988

0.554910004 0.073820002 0.759809971

0.510749996 0.966279924 0.711150050

0.597859979 0.846679986 0.516080022

0.501580000 0.759079993 0.568030000

0.887070000 0.437330008 0.203259990

0.806819975 0.456079960 0.143240005

0.710489988 0.773980021 0.571560025

0.825049996 0.740339994 0.554979980

0.498730004 0.592270017 0.157140002

0.083640009 0.247100011 0.724950016

0.182490006 0.230519995 0.683250010

0.811389983 0.057539999 0.720049977

0.836950004 0.209950000 0.703779936

0.172869995 0.284560025 0.597920001

0.193240002 0.356929988 0.541059971

0.611429989 0.293119997 0.966759920

0.516529977 0.219260022 0.915310025

0.000240001 0.153640002 0.340229988

0.881429970 0.162349999 0.372299999

0.101280026 0.871180058 0.882780015

0.058220003 0.729529977 0.918920040

0.663489997 0.936339974 0.306670010

0.750670016 0.017099999 0.252249986

0.547399998 0.853259981 0.228619993

0.481090009 0.821959972 0.165319994

0.225229993 0.012540000 0.315880001

0.369049996 0.081939995 0.284869999

0.242100000 0.079810001 0.515860021

0.143820003 0.160270020 0.502330005

0.147350013 0.612389982 0.368499994

0.097320005 0.711539984 0.335079998
