# Supplementary material for: Ampere-level reduction of pure nitrate by electron-deficient Ru with K+ ions repelling effect
Source: Nat Commun. 2024 Dec 30;15:10877. doi: 10.1038/s41467-024-55230-w (PMC11685401; doi:10.1038/s41467-024-55230-w)
Supplement: Supplementary file 6 — Supplementary Data 4 [file 41467_2024_55230_MOESM6_ESM.docx]

POSCAR file written by OVITO Basic 3.7.6

1.0

10.8232002258 0.0000000000 0.0000000000

-5.4116001129 9.3731663458 0.0000000000

0.0000000000 0.0000000000 23.3885002136

Ru O N K H

16 61 2 3 109

Direct

0.083329998 0.166669995 0.057290003

0.333330005 0.166669995 0.057290003

0.583329976 0.166669995 0.057290003

0.833329976 0.166669995 0.057290003

0.083330013 0.416669995 0.057290003

0.333330005 0.416669995 0.057290003

0.583329976 0.416669995 0.057290003

0.833329976 0.416669995 0.057290003

0.083330013 0.666670024 0.057290003

0.333330005 0.666670024 0.057290003

0.583329976 0.666670024 0.057290003

0.833329976 0.666670024 0.057290003

0.083329976 0.916670024 0.057290003

0.333330005 0.916670024 0.057290003

0.583329976 0.916670024 0.057290003

0.833329976 0.916670024 0.057290003

0.454175770 0.168841809 0.930322409

0.924905002 0.096042611 0.737252772

0.677046537 0.236260533 0.196439087

0.588481009 0.671240747 0.969177783

0.996617436 0.799491763 0.919653714

0.352569729 0.938563645 0.153384238

0.387391925 0.328940392 0.726015270

0.604771554 0.174549520 0.845575452

0.646817565 0.998381734 0.781025529

0.068571344 0.849418581 0.479596794

0.990645766 0.442309171 0.196168855

0.865756810 0.056637645 0.318167210

0.651607931 0.874415636 0.387975752

0.782677948 0.726245761 0.726226032

0.705895543 0.780457675 0.837369204

0.104450345 0.529838681 0.923122585

0.289162189 0.134457409 0.637339830

0.023045845 0.636947215 0.330952287

0.888292074 0.316642910 0.686641514

0.129765809 0.012665140 0.275400877

0.312422127 0.902537107 0.965035081

0.335463434 0.211039022 0.833764672

0.676590741 0.211876869 0.321899742

0.860318542 0.949953914 0.965657473

0.722553492 0.460632354 0.808413506

0.435252041 0.956767321 0.595995545

0.227009520 0.724973798 0.528328776

0.386644870 0.801331699 0.427961946

0.533460379 0.538102031 0.378846228

0.435956359 0.240356445 0.434190750

0.996499300 0.606512666 0.833077967

0.691898227 0.442826331 0.691450417

0.818107665 0.912229717 0.145244002

0.333546400 0.409480631 0.968433380

0.203976572 0.176841229 0.528260410

0.403655261 0.727610767 0.913469255

0.034512822 0.783315241 0.208299413

0.721030593 0.353482783 0.461983591

0.824284256 0.631143570 0.406381875

0.528381288 0.856772721 0.675445437

0.198848277 0.912760556 0.374478668

0.619869828 0.492795438 0.192703068

0.556823611 0.678347170 0.489690542

0.582359314 0.683532476 0.143761650

0.793089330 0.913666189 0.646541953

0.057487607 0.906172812 0.705464125

0.987044275 0.422866881 0.575142026

0.680967450 0.022165811 0.919617057

0.408161223 0.561672032 0.273028761

0.032544434 0.248764887 0.905138493

0.725794554 0.002337456 0.222423553

0.437349707 0.776827514 0.208217159

0.968557775 0.215880543 0.508364141

0.721472502 0.962299645 0.535221875

0.130749986 0.463893086 0.313161701

0.307152063 0.396175921 0.142455354

0.112633333 0.175976828 0.142669231

0.222416803 0.280444413 0.227219746

0.223044321 0.474056065 0.810429275

0.360907942 0.575210214 0.733842909

0.179965451 0.606109560 0.750301838

0.256301343 0.551804364 0.766077697

0.216497108 0.284448773 0.169902667

0.022536993 0.035099350 0.832882226

0.507241011 0.480857372 0.863540947

0.922538221 0.295806944 0.387713790

0.500357568 0.213494122 0.969835639

0.531157255 0.179010123 0.903373539

0.921086609 0.177368626 0.718988895

0.904203951 0.038457211 0.704409480

0.654444456 0.199802920 0.154998839

0.773526967 0.312793136 0.192940339

0.677986622 0.752157807 0.959124744

0.505070508 0.684968114 0.955800176

0.005533738 0.742002130 0.889302492

0.973104179 0.750444651 0.957688093

0.390449524 0.875131249 0.168818474

0.403349549 0.037266538 0.167651042

0.350797415 0.264312178 0.691770375

0.354948968 0.401589930 0.723878145

0.630088508 0.119299613 0.816079795

0.650252581 0.276091188 0.829019070

0.740224838 0.042025428 0.762286127

0.579722643 0.946826696 0.748893499

0.116247863 0.897732258 0.440129936

0.973998725 0.769212544 0.468406320

0.007388197 0.536276340 0.194400653

0.016538206 0.429702759 0.156635940

0.949230790 0.043394089 0.311583608

0.814761937 0.026787281 0.278691292

0.734647155 0.932767928 0.363015771

0.668661535 0.818732858 0.413726747

0.777614653 0.772153854 0.690876305

0.746205568 0.630170286 0.708842397

0.680869520 0.856069982 0.831685960

0.723141491 0.757282376 0.797322690

0.102918744 0.563180625 0.961052477

0.203960776 0.587338209 0.912275255

0.272245288 0.168895200 0.600336790

0.192894205 0.078697748 0.656122804

0.007250933 0.660302997 0.291524857

0.071016274 0.578562200 0.323808372

0.923892796 0.391685039 0.714591205

0.931148708 0.364649028 0.649342358

0.159761608 0.097611643 0.253106296

0.083160900 0.936203539 0.245750070

0.215413898 0.852258861 0.949542522

0.362971127 0.011577010 0.952492654

0.390541881 0.165262610 0.833366632

0.347177863 0.246261612 0.793387532

0.588348746 0.118683457 0.328413934

0.702099204 0.218271732 0.281768858

0.811813056 0.992275536 0.938728988

0.898233354 0.893830657 0.946698904

0.829071641 0.510851085 0.822063625

0.722699165 0.478741258 0.766558349

0.399532259 0.019144291 0.610776782

0.351655304 0.872606933 0.580478847

0.178260908 0.785696387 0.518365324

0.294395834 0.743290961 0.495857954

0.479273319 0.836498976 0.409075946

0.339893281 0.846437097 0.405470103

0.474480093 0.544224858 0.348935366

0.473634899 0.444366217 0.398101956

0.542904258 0.302827001 0.448499143

0.425141722 0.161407709 0.409984171

0.063384295 0.613599002 0.801683486

0.030176878 0.561222851 0.863090515

0.590606987 0.381089956 0.692104161

0.736507833 0.382328629 0.688431859

0.679583073 0.762551308 0.149772704

0.920024574 0.962496817 0.157324880

0.342099905 0.329666436 0.957076311

0.245518506 0.394684821 0.952551126

0.149606392 0.075633340 0.521425307

0.279356301 0.204667166 0.498866826

0.384142697 0.786161780 0.943022907

0.462220401 0.807584643 0.888609409

0.127341986 0.826295197 0.188989252

0.956442595 0.747756004 0.182252035

0.756167531 0.362945467 0.499952614

0.753501713 0.454676449 0.446951151

0.733300090 0.616078079 0.387521982

0.895992041 0.638912976 0.376145691

0.460871458 0.762016118 0.680721641

0.492376447 0.899701655 0.643120050

0.203218952 0.977347136 0.344131768

0.134340510 0.811780274 0.358722925

0.606754661 0.447087288 0.230380699

0.618256271 0.427148879 0.159958348

0.546688914 0.636438131 0.450514466

0.474006325 0.685816288 0.495427370

0.582572103 0.596526802 0.162631854

0.691551089 0.888206601 0.654103160

0.810397446 0.952432394 0.607029974

0.959640741 0.832887650 0.715318382

0.099771261 0.847430527 0.695229948

0.057566229 0.508892238 0.556820333

0.977001905 0.345225096 0.550190151

0.691142499 0.107622854 0.900365293

0.631217837 0.010636570 0.956070602

0.403939307 0.493757010 0.244353980

0.401837647 0.641776443 0.251107305

0.993041635 0.255353868 0.943531215

0.064883851 0.347253323 0.894294083

0.722341001 0.092705846 0.216776490

0.760655463 0.986018419 0.183602557

0.537559152 0.852648497 0.220848545

0.457081050 0.718317389 0.181160465

0.879240274 0.119447842 0.507509172

0.053043060 0.207501262 0.514420807

0.673016667 0.858451366 0.522661209

0.636982918 0.977604210 0.538178563

0.229481220 0.512843549 0.301949292

0.089613728 0.438029468 0.273147792
